# Poised Transcription Factories Prime Silent uPA Gene Prior to Activation

**DOI:** 10.1371/journal.pbio.1000270

**Published:** 2010-01-05

**Authors:** Carmelo Ferrai, Sheila Q. Xie, Paolo Luraghi, Davide Munari, Francisco Ramirez, Miguel R. Branco, Ana Pombo, Massimo P. Crippa

**Affiliations:** 1 Laboratory of Molecular Dynamics of the Nucleus, Division of Genetics and Cell Biology, S. Raffaele Scientific Institute, Milan, Italy; 2 Medical Research Council Clinical Sciences Centre, Imperial College School of Medicine, Hammersmith Hospital Campus, London, United Kingdom; 3 South Ruislip, Middlesex, United Kingdom; National Cancer Institute, United States of America

## Abstract

The position of genes in the interphase nucleus and their association with functional landmarks correlate with active and/or silent states of expression. Gene activation can induce chromatin looping from chromosome territories (CTs) and is thought to require de novo association with transcription factories. We identify two types of factory: “poised transcription factories,” containing RNA polymerase II phosphorylated on Ser5, but not Ser2, residues, which differ from “active factories” associated with phosphorylation on both residues. Using the urokinase-type plasminogen activator (uPA) gene as a model system, we find that this inducible gene is predominantly associated with poised (S5p^+^S2p^−^) factories prior to activation and localized at the CT interior. Shortly after induction, the uPA locus is found associated with active (S5p^+^S2p^+^) factories and loops out from its CT. However, the levels of gene association with poised or active transcription factories, before and after activation, are independent of locus positioning relative to its CT. RNA-FISH analyses show that, after activation, the uPA gene is transcribed with the same frequency at each CT position. Unexpectedly, prior to activation, the uPA loci internal to the CT are seldom transcriptionally active, while the smaller number of uPA loci found outside their CT are transcribed as frequently as after induction. The association of inducible genes with poised transcription factories prior to activation is likely to contribute to the rapid and robust induction of gene expression in response to external stimuli, whereas gene positioning at the CT interior may be important to reinforce silencing mechanisms prior to induction.

## Abstract 2

The association of poised genes with transcription factories may contribute to rapid transcriptional activation in response to stimuli and to silencing when genes are located at the interior of their chromosome territories.

## Author Summary

The spatial organization of the genome inside the cell nucleus is important in regulating gene expression and in the response to external stimuli. Examples of changing spatial organization are the repositioning of genes outside chromosome territories during the induction of gene expression, and the gathering of active genes at transcription factories (discrete foci enriched in active RNA polymerase). Recent genome-wide mapping of RNA polymerase II has identified its presence at many genes poised for activation, raising the possibility that such genes might associate with poised transcription factories. Using an inducible mammalian gene, urokinase-type plasminogen activator (uPA), and a system in which this gene is poised for expression, we show that uPA associates with poised transcription factories prior to activation. Gene activation induces two independent events: repositioning towards the exterior of its chromosome territory and association with active transcription factories. Surprisingly, genes inside the interior of the chromosome territory prior to activation are less likely to be actively transcribed, suggesting that positioning at the territory interior has a role in gene silencing.

## Introduction

The spatial folding of chromatin within the mammalian cell nucleus, from the level of whole chromosomes down to single genomic regions, is thought to contribute to the expression status of genes [1]–[3]. Mammalian chromosomes occupy discrete domains called chromosome territories (CTs) and have preferred spatial arrangements within the nuclear landscape in specific cell types, which are conserved through evolution [1]–[3]. Subchromosomal regions containing inducible genes, such as the MHC type II or Hox gene clusters, relocate outside their CTs upon transcriptional activation or when constitutively expressed [4],[5]. Genes can preferentially associate with specific nuclear domains according to their expression status. Most noteworthy, gene associations with the nuclear lamina largely correlate with silencing [6]–[9], whereas gene associations with transcription factories, discrete clusters containing many RNA polymerase II (RNAP) enzymes, have been observed only when genes are actively transcribed, but not during the intervening periods of inactivity [2].

Although CTs do not represent general barriers to the transcriptional machinery [10],[11] and transcription can occur inside CTs [3],[12]–[14], the large-scale movements of chromatin, observed in response to gene induction, have often been interpreted as favouring gene associations with compartments permissive for transcription [15]–[17]. However, inducible genes frequently display an active chromatin configuration and are primed by initiation-competent RNAP complexes prior to induction [18]–[21].

Complex phosphorylation events at the C-terminal domain (CTD) of the largest subunit of RNAP correlate with initiation and elongation steps of the transcription cycle and are crucial for chromatin remodelling and RNA processing [22],[23]. The mammalian CTD is composed of 52 repeats of an heptad consensus sequence Tyr^1^-Ser^2^-Pro^3^-Thr^4^-Ser^5^-Pro^6^-Ser^7^, and phosphorylation on Ser^5^ residues (S5p) is associated with transcription initiation and priming, whereas phosphorylation on Ser^2^ (S2p) correlates with transcriptional elongation [22],[23].

To investigate whether primed genes are associated with discrete RNAP sites enriched in RNAP-S5p and the functional relevance of large-scale gene repositioning in promoting associations with the transcription machinery during gene activation, we investigated the expression levels, epigenetic status, nuclear position, and association with RNAP factories of an inducible gene, the urokinase-type plasminogen activator (uPA or PLAU; GeneID 5328), before and after activation. We use antibodies that specifically detect different phosphorylated forms of RNAP to investigate the association of the inducible uPA gene with transcription factories. Prior to induction, most uPA alleles are positioned inside their CT and extensively associated with RNAP sites marked by S5p. Transcriptional activation leads to looping out of the uPA locus from its CT, and increased association with active transcription factories marked by both S5p and S2p. However, the extent of gene association with factories, before and after activation, is independent of the uPA position relative to its CT. Unexpectedly, we find that the majority of uPA genes which are positioned at the CT interior prior to activation are seldom transcribed, in comparison with the few uPA genes located outside the CT which are active with the same frequency as the fully induced uPA genes.

## Results/Discussion

### Transcriptional Induction of the uPA Locus Promotes Relocation Outside Its CT

**Figure 1:**
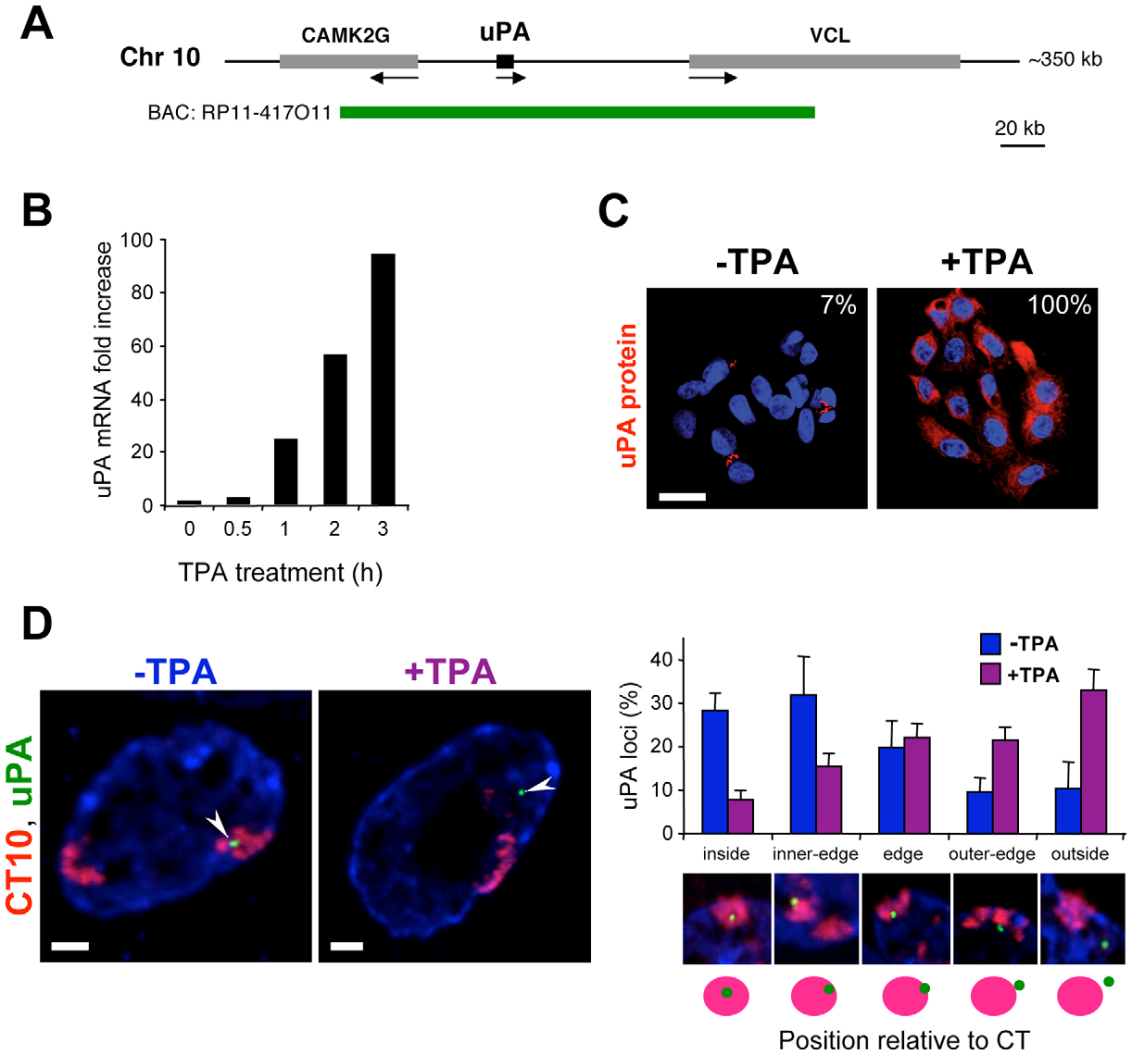
Activation of the uPA gene by TPA treatment induces large-scale chromatin repositioning in the nucleus of HepG2 cells. (A) Diagram illustrating the genomic context of the uPA locus and the genomic region detected by the BAC probe (RP11-417O11; ∼228 kb) used for FISH experiments. CAMK2G, calcium-calmodulin-dependent protein kinase II gamma; VCL, vinculin. Arrows indicate the 5′-3′ transcription direction. (B) Kinetics of the transcriptional induction of the uPA gene with TPA. uPA RNA expression was assessed by quantitative RT-PCR after treatment with TPA for different times. Values were normalized to 18S rRNA and expressed relative to uninduced cells. (C) Induction of uPA protein expression with TPA. Indirect immunofluorescence analyses of uPA protein expression (red) before and after TPA treatment (3 h). Nuclei were counterstained with DAPI (blue). The proportion of uPA-expressing cells is indicated (*n* = 188 and 144 for untreated and treated cells, respectively). Bar: 20 µm. (D) TPA induces large-scale repositioning of the uPA locus (green) to the exterior of its own chromosome 10 territory (CT10; red). The position of the uPA locus relative to CT10 was determined in HepG2 cells, before and after TPA activation for 3 h, by cryoFISH using a whole chromosome 10 paint (red) and the digoxigenin-labelled BAC probe (green) represented in (A). Nucleic acids were counterstained with TOTO-3 (blue). Arrowheads indicate uPA loci. The positions of uPA loci relative to the CT were scored as “inside,” “inner-edge,” “edge,” “outer-edge,” and “outside”; error bars represent standard deviations. Bars: 2 µm.

The uPA gene encodes a serine protease that promotes cell motility, and its overexpression is known to correlate with cancer malignancies and tumor invasion [24]–[26]. It is a 6.4 kb gene with 10 introns, and its regulatory regions have been extensively characterized [24]. uPA is located on human chromosome 10, separated from upstream and downstream flanking genes CAMK2G (calcium calmodulin-dependent protein kinase II gamma; GeneID 818) and VCL (vinculin; GeneID 7414) by ∼40 and ∼80 kb, respectively (Figure 1A). In HepG2 cells, where the uPA gene is present as a single copy, its transcription can be induced through various stimuli, including treatment with phorbol esters [27]. Tetradecanoyl phorbol acetate (TPA) induces its expression by ∼100-fold in HepG2 cells after 3 h of treatment (Figure 1B). The induction of the uPA gene within this short time of activation occurs in all cells of the population, as shown by immunofluorescence detection of uPA protein in single cells (Figure 1C); low levels of uPA protein are detected in a small proportion (7%) of the cell population prior to activation.

We first investigated whether transcriptional induction of the uPA gene was associated with large-scale repositioning relative to its CT, using a whole chromosome 10 probe together with a BAC probe containing the uPA locus (Figure 1A). We performed fluorescence in situ hybridization on ultrathin (∼150 nm) cryosections (cryoFISH), a method that preserves chromatin structure and organisation of transcription factories. Cells are fixed using improved formaldehyde fixation in comparison with standard 3D-FISH, which is particularly important for the preservation of chromatin structure and RNAP distribution [14],[28]. CryoFISH also provides sensitivity of detection and high spatial resolution, especially in the *z* axis [14],[29],[30].

We find that, in the inactive state, the uPA locus is preferentially localized at the CT interior (60% loci inside or at the inner-edge, *n* = 166 loci) and relocates to the exterior upon activation (55% loci at outer-edge or outside, *n* = 208 loci; χ^2^ test, *p*<0.0001; Figure 1D), concomitant with the 100-fold induction of mRNA levels determined by qRT-PCR (Figure 1B). Thus, we observed a striking change in the position of the uPA locus relative to its CT upon TPA activation, which correlates with a major increase in mRNA and protein expression across the whole population of cells.

### Induction of the uPA Locus and Its Nuclear Relocation Are Independent of Local Chromatin Decondensation

**Figure 2:**
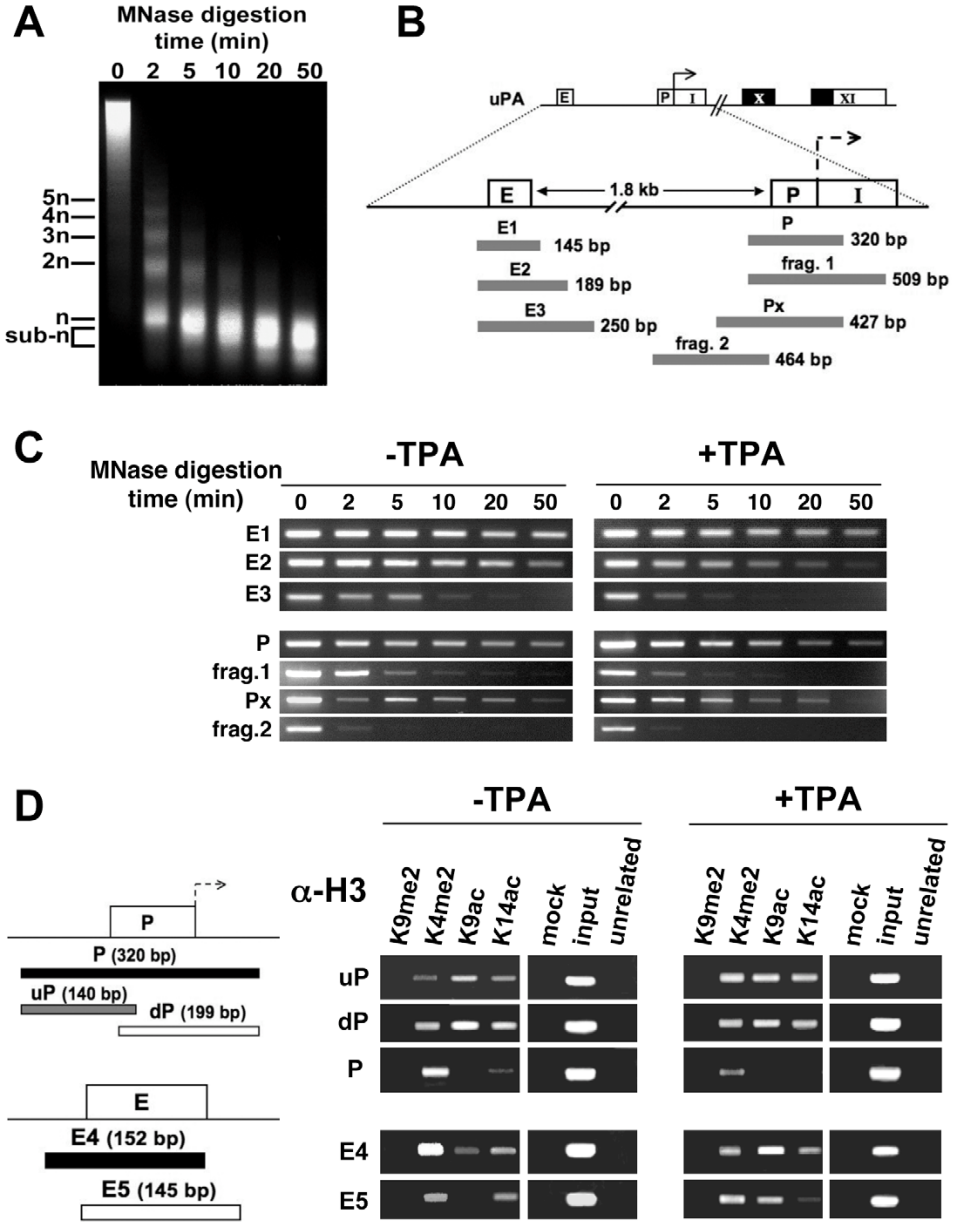
The regulatory regions of the uPA locus display an open chromatin conformation before and after TPA activation. (A) Micrococcal nuclease (MN) digestion progressively cleaves cross-linked, sonicated bulk chromatin to mononucleosomes. Material from the 50 min digestion time-point was used for MN-ChIP experiments. (B) Diagram illustrating the regulatory region of the uPA gene, including the enhancer (E), promoter (P), and the position of amplified genomic fragments at and around the enhancer and promoter regions. Roman numerals indicate exons in the coding region (white and black boxes represent untranslated and translated regions, respectively). (C) Chromatin-associated features of the uPA enhancer and promoter regions as revealed by PCR amplification patterns of MN-digested chromatin DNA before and after transcriptional activation. HepG2 cells were grown ±TPA for 3 h, before chromatin preparation. Enhancer fragment 1 (E1), but not E2 and E3, is resistant to MNase cleavage after 50 min of digestion. Two promoter fragments larger than a single nucleosome (P and Px, 320 bp and 464 bp, respectively) are detected at the same digestion time point in the promoter region, consistent with protection due to additional bound proteins such as transcription factors and RNAP [31]. (D) MN-ChIP experiments identify histone modifications associated with open chromatin at the enhancer and promoter regions before and after TPA activation. Chromatin was cross-linked, sonicated, and treated with MN for 50 min, prior to immunoprecipitation with antibodies against specific histone modifications associated with open (H3K4me2, H3K9ac, H3K14ac) or closed (H3K9me2) chromatin. Control immunoprecipitations were performed with polyclonal anti-uPA receptor (uPAR) antibodies (unrelated). To improve the resolution of MN-ChIP in the promoter region, two smaller, overlapping genomic fragments spanning the entire P fragment (uP and dP, 140 and 199 bp, respectively) were also amplified.

Chromatin repositioning in response to gene activation has often been associated with changes in chromatin structure and degree of condensation [15],[17]. To establish whether the large-scale relocation of the uPA locus during its transcriptional activation was accompanied by changes between closed and open chromatin conformations, we next assessed the chromatin structure of the uPA gene before and after TPA treatment (Figure 2). Micrococcal nuclease (MN) digestion of crosslinked, sonicated chromatin yields a decreasing nucleosomal ladder before and after TPA activation (Figure 2A and images unpublished, respectively; [31]). Systematic PCR amplification at and around the uPA regulatory regions revealed two populations of genomic DNA fragments that resist processive cleavage at high digestion time points (50 min; see also [31]). At the enhancer, the size of fragments is typically mononucleosomal (∼150 bp; fragment E1; Figure 2B, 2C). At the promoter, the protected fragments have larger sizes (>300 bp; fragments P and Px; Figure 2B, 2C). This feature is consistent with the presence of RNAP-containing complexes at the promoter, which was previously observed at the transcriptionally active uPA gene in constitutively expressing cells, but absent after α-amanitin treatment [31]. The same population of larger promoter fragments was also detected in uninduced cells (Figure 2C), showing that the uPA gene displays transcription-associated features before activation. This was supported by an investigation of the epigenetic status of chromatin before and after activation. High resolution, MN-coupled chromatin immunoprecipitation (MN-ChIP; [31]) using antibodies specific for histone modifications associated with close (H3K9me2) or open (H3K4me2, H3K9ac, H3K14ac) chromatin [32] showed the presence of active, but not silent, chromatin marks at the promoter and enhancer of the uPA gene, both before and after TPA induction (Figure 2D). Positive detection of H3K9me2 was confirmed at the imprinted H19 gene (GeneID 283120; Figure S1). Upon activation, the larger promoter fragment (P) is no longer detected with H3K14ac antibodies, although this mark is still present at the smaller promoter fragments (uP and dP), and an enrichment of H3K9ac at the E5 fragment on the enhancer was also detected (Figure 2D). These changes are likely to reflect the presence of different populations of resistant fragments at the uPA regulatory regions upon induction. Taken together, these findings show that the uPA gene adopts an open chromatin state before transcriptional activation, which is maintained after induction. The large-scale chromatin repositioning of the uPA locus relative to its CT (Figure 1D) cannot therefore be explained by changes from closed to open chromatin conformation.

### Inactive uPA Genes Are Associated with Poised Transcription Factories Prior to Induction

The presence of RNAP phosphorylated on Ser5 residues at promoter regions of silent genes defines them as paused or poised genes [21]–[23]. To determine whether the inactive uPA gene was associated with RNAP prior to induction, we used MN-ChIP and antibodies specific for phosphorylated forms of RNAP that can discriminate between active and paused/poised RNAP complexes [21]–[23]. Antibody specificity has been extensively characterized previously [21],[33] and was confirmed in HepG2 cells using Western blotting and immunofluorescence (Figure S2).

**Figure 3:**
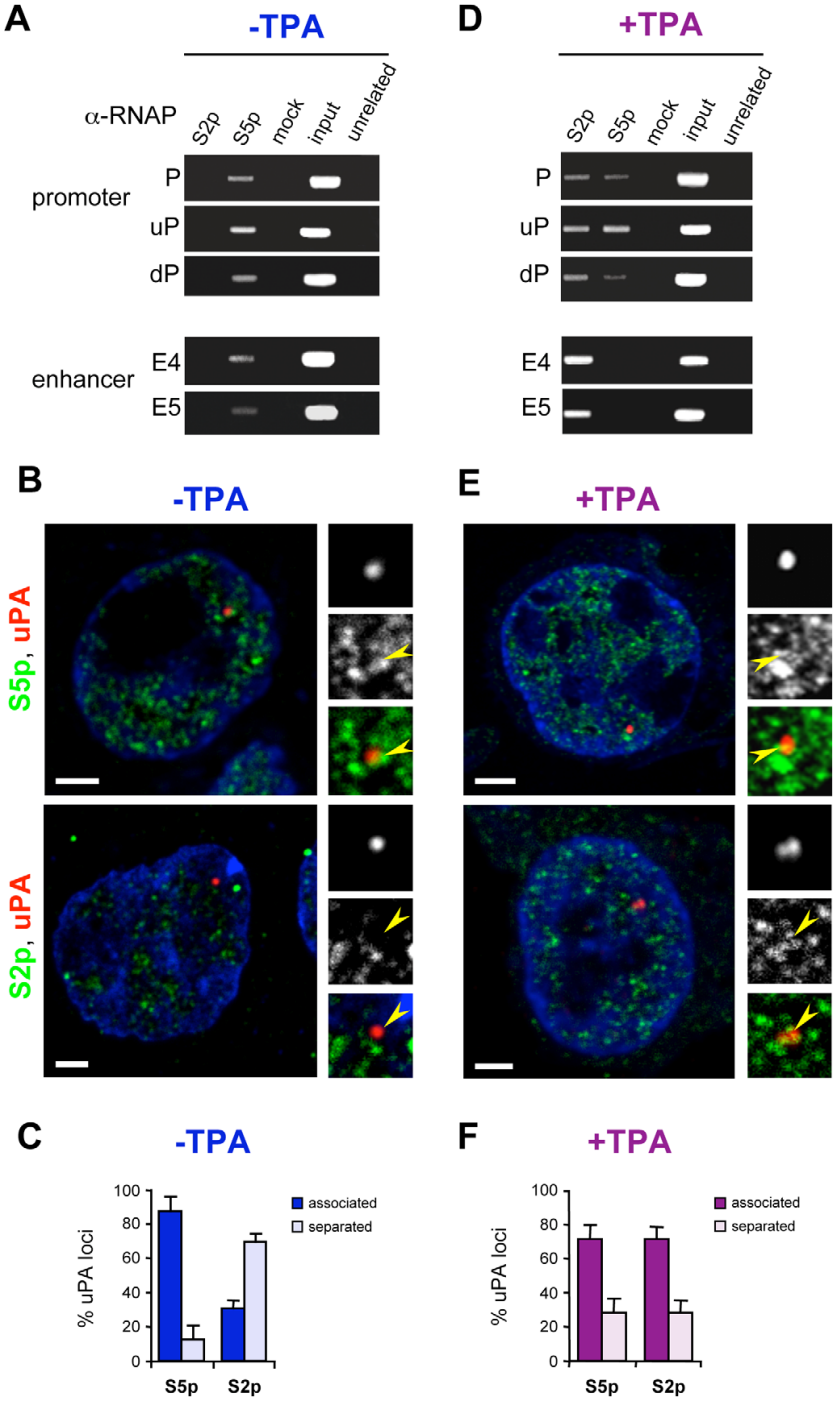
Inactive uPA loci associate with poised transcription factories rich in the initiating form of RNAP (S5p), prior to activation. (A, D) MN-ChIP analyses detect the initiating (S5p) form of RNAP bound at the uPA promoter and enhancer before TPA activation (A). Following activation the enhancer is associated with the elongating form of RNAP (S2p), while the promoter is associated with both forms (S5p and S2p) of the enzyme (D). HepG2 cells were grown ±TPA for 3 h, before chromatin preparation and MN-ChIP with antibodies specific for S5p and S2p forms of RNAP. Control (unrelated) antibodies were polyclonal anti-uPA receptor antibodies. (B, E) The position of the uPA locus (red) relative to S5p and S2p sites (green) was determined by immuno-cryoFISH before (B) and after (E) TPA activation for 3 h, using a rhodamine-labelled BAC probe containing the uPA locus and antibodies specific for RNAP phosphorylated on S5 or S2 residues. The association of uPA genes with RNAP (S5p or S2p) was scored as “associated” (signals overlap by at least 1 pixel) or “separated” (signals do not overlap or are adjacent; see Figure S8 for additional examples). Arrowheads indicate the position of uPA loci. Nucleic acids were counterstained with TOTO-3 (blue). Bars: 2 µm. (C, F) Frequency of association of uPA loci with RNAP-S5p or -S2p sites before (C) and after (F) TPA activation. The decrease in association with S5p factories and the increase in association with S2p factories observed after activation were both statistically significant (χ^2^ tests, *p* = 0.0006 and *p*<0.0001, respectively).

MN-ChIP detected the initiating (S5p) but not the elongating (S2p) form of RNAP at the promoter and enhancer regions of the uPA gene prior to induction (Figure 3A), demonstrating that the uPA gene is primed with RNAP before activation. The detection of RNAP-S5p at the enhancer (Figure 3A) can be explained by an interaction with RNAP bound at the promoter, as previously observed in constitutively active uPA genes [31].

Previous studies describing the presence of RNAP at the promoters of paused or poised genes did not investigate a possible association with transcription factories marked specifically by the S5p modification [18],[19],[21],[34]. We asked whether the association of primed uPA loci with RNAP-S5p could be detected at the single cell level and occur within specific nuclear substructures, using immuno-cryoFISH [14]. Using a BAC probe covering a genomic region centred on the uPA gene (Figure 1A) in combination with immunolabelling of the S5p or S2p forms of RNAP, we found that the vast majority of uPA loci were associated with sites containing RNAP-S5p prior to activation (87%±9%, *n* = 165 loci; Figure 3B, 3C). A significantly lower proportion was found associated with RNAP-S2p foci (31%±5%, *n* = 170 loci; χ^2^ test, *p*<0.0001; Figure 3C). The primed uPA loci are therefore preferentially associated with a subpopulation of RNAP factories that contain RNAP-S5p, but not RNAP-S2p, prior to TPA activation. We call these sites “poised,” or S5p^+^S2p^−^, transcription factories.

Scoring criteria for gene association with RNAP sites, typically used in the analyses of 3D-FISH results, often rely on proximity criteria that do not involve true physical associations, being sensitive to the limited z axis resolution (>500 nm) of standard confocal microscopes. This is particularly important when analysing highly abundant structures such as transcription factories which can exist at densities of 20/µm^3^ [35]. Although the use of ultrathin (∼150 nm) cryosections mostly detects single factories [29], we were still concerned that the extent of uPA gene association with transcription factories marked by S5p or S2p observed experimentally (Figure 3C) could be due to different abundance of the two modifications and might be explained, at least in part, by random processes. To assess the impact of these two constraints, we generated one simulated uPA signal, for each experimental image, with the same number of pixels as the experimental site, but positioned at random coordinates within the nucleoplasm. Next, we measured the frequency of association of the randomly positioned loci with RNAP-S5p or -S2p sites (Figure S3B, S3C). We found that the association of randomly positioned BAC signals with S5p was 54%±8% (Figure S3C; *n* = 68 loci), a significantly lower number than the experimental value of 87%±9% for the uPA locus (Figure 3C; χ^2^ test, *p*<0.0001). In contrast, the association of randomly positioned signals with S2p was 39%±5% (*n* = 69 loci), similar to the experimental value of 31%±5% (χ^2^ test, *p* = 0.29; Figure S3C). One caveat of these analyses is that the observation of similar levels of association for experimental or simulated loci with transcription factories prior to activation cannot be used to argue that this association is not specific, but simply that it is as low as it would be if loci were positioned randomly. In summary, our results show that the association of a large proportion of uPA loci with poised, S5p^+^S2p^−^ factories before activation is specific, although the nuclear environment where the uPA loci are located is not devoid of active, S5p^+^S2p^+^ transcription factories, and therefore seems to be permissive for transcription.

### The uPA Gene Associates with Active Transcription Factories upon Activation

To investigate the active state of the uPA gene and the engagement of the locus with active factories following transcriptional induction, we repeated the MN-ChIP and immuno-cryoFISH analyses for TPA-treated cells (Figure 3D–F). MN-ChIP showed that the enhancer and the promoter of the uPA gene are associated with the elongating (S2p) form of RNAP either together with RNAP-S5p (at the promoter) or exclusively (at the enhancer; Figure 3D). The absence of RNAP-S5p at the enhancer fragments analysed, in the presence of S2p, suggests that the enhancer may maintain an association with RNAP as it moves through the coding region during elongation, where S5p is known to decrease and S2p to augment [23]. Immuno-cryoFISH after TPA induction (Figure 3E, 3F) showed that the activated uPA locus now becomes associated with RNAP-S2p sites (72%±7% loci, *n* = 183), while maintaining an association with RNAP-S5p (71%±8%, *n* = 140 loci; χ^2^ test, *p* = 0.98), consistent with the MN-ChIP results. The approximately 2-fold increase in association with RNAP-S2p from 31% to 72%, before and after TPA treatment, respectively, was highly statistically significant (χ^2^ test, *p*<0.0001). Evaluation of simulated uPA loci positioned at random coordinates in the same experimental images showed that the increased association of the uPA locus with S2p observed after activation (72%) cannot be explained by random processes, as the frequency of association of simulated loci remained at 38%±2% (*n* = 75 loci; χ^2^ test, *p*<0.0001; Figure S3B, S3C). An increased association with S2p sites upon activation has also recently been described for the *Hoxb*1 gene in mouse embryonic stem cells, albeit at lower frequency [36].

Taken together we show that the activation of the uPA gene and large-scale repositioning of the locus relative to its CT coincide with the acquisition of the S2p modification of RNAP without major changes in chromatin structure.

### Factories Containing S2p Are Also Marked by the S5p Modification and Are Less Abundant Than Sites Marked by S5p

**Figure 4:**
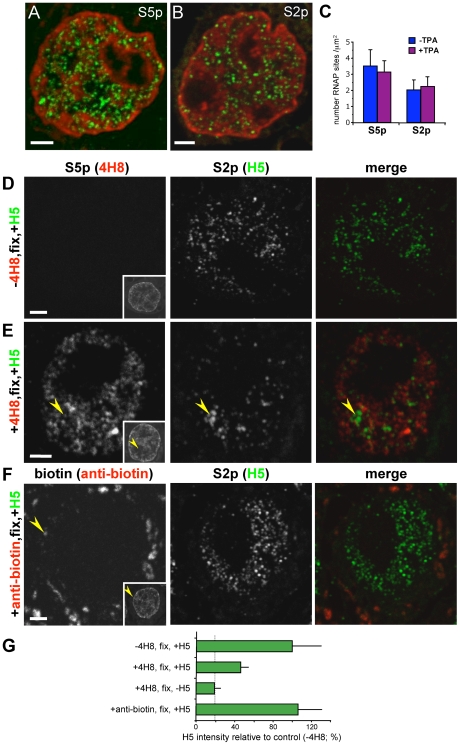
Factories containing S2p are also marked by the S5p modification and are less abundant than sites marked by S5p. (A–C) RNAP-S5p sites are more abundant than RNAP-S2p sites. Cryosections (∼140 nm thick) from HepG2 cells grown ±TPA for 3 h were indirectly immunolabelled with antibodies specific for RNAP-S5p or -S2p (green), as indicated (A, B). Nuclei were counterstained with TOTO-3 (red). Representative images from TPA-treated cells are shown. RNAP-S5p and -S2p detection was optimised by using the highest concentrations of antibodies that give little detectable background in sections treated with alkaline phosphatase (see Figure S2E, S2F). Bars: 2 µm. Measurement of the number of S5p and S2p sites per unit area in the nucleoplasm (C) reveals a larger population of S5p than S2p sites, both before and after activation (Student *t*-test, *p*<0.0001 for both cases; number of nuclear sections analysed were for S5p, *n* = 45 and 41, and for S2p, *n* = 38 and 46, respectively, for − and +TPA). The decrease in S5p sites after TPA activation is statistically significant (*p* = 0.012), whereas no statistically significant difference was observed in the number of S2p sites (*p* = 0.44). (D–F) Most S2p sites also contain the S5p modification. Cryosections were first indirectly immunolabelled in the absence (D) or presence (E) of an antibody against RNAP-S5p (4H8; red) or in the presence of an unrelated anti-biotin antibody (F; red) that detects mitochondria in the cytoplasm (F; arrowheads). After formaldehyde cross-linking to preserve the first immunocomplex, sections were indirectly immunolabelled with an antibody against RNAP-S2p (H5; green). Nucleic acids were counterstained with TOTO-3 (insets), and confocal images collected using the same settings without signal saturation. Bars: 2 µm. Pre-incubation with 4H8 reduces the intensity of S2p signal throughout the nucleoplasm, except at interchromatin regions (E, arrows), in comparison to control samples incubated in the absence of 4H8 (D). Incubation with anti-biotin control antibody before labelling with H5 antibody (F) has no effect on S2p distribution throughout the nucleoplasm. (G) Measurements of average S2p intensity across the nucleoplasm show a ∼3-fold decrease in S2p detection after blocking with 4H8. Omission of 4H8 or pre-incubation with unrelated antibodies does not affect the level of the S2p signal in the nucleoplasm. Dotted line indicates the background intensity in the S2p channel measured from sections incubated with all antibodies except H5 (−H5; images unpublished). Number of nuclear profiles was >20 for each sample.

The striking agreement between the number of uPA loci associated with RNAP factories marked by S5p and S2p upon activation and the co-existence of the two RNAP modifications detected by MN-ChIP at the promoter suggest that active factories contain both modifications, as expected from concomitant initiation and elongation events at promoter and coding regions of highly active genes. To further investigate whether poised transcription factories marked by S5p alone are distinct from the active factories marked by S2p, we compared the number of RNAP-S5p and RNAP-S2p sites in HepG2 cells, before and after induction (Figure 4A–C).

RNAP sites marked by S5p are significantly more abundant than RNAP sites marked by S2p (28% excess) both before and after activation (Student *t* test, *p*<0.0001 in both cases; Figure 4A–C), suggesting that a considerable number of transcription factories adopt the poised state. The excess number of sites containing S5p in the absence of S2p (Figure 4A–C) is consistent with recent reports identifying an abundance of primed genes [34],[37]–[39] marked by RNAP-S5p and not RNAP-S2p [21] in embryonic stem cells or differentiated cells.

To investigate to what extent S2p sites are also marked by S5p, we used an antibody-blocking assay ([30],[40]; Figure 4D–G), in which sections were first incubated with antibodies against RNAP-S5p before incubation with antibodies against RNAP-S2p. Simultaneous incubation with the two antibodies resulted in a ∼65% quenching of the detection of RNAP-S2p (images unpublished), due to a more efficient binding of 4H8, an IgG, in comparison with H5, an IgM. The rationale of antibody blocking experiments is that the binding of the first antibody prevents binding by the second, if the two respective epitopes are located within the distance corresponding to the size of the first bound antibody complex (Ser2 and Ser5 residues are separated by two aminoacids whereas IgGs are large proteins that measure ∼9 nm). After pre-incubation with the specific S5p antibody (4H8), the overall intensity of RNAP-S2p sites was significantly reduced throughout the nucleoplasm, except in discrete interchromatin domains (Figure 4E, 4G), as compared to sections not incubated with this antibody (Figure 4D, 4G) or to sections incubated with an unrelated (anti-biotin) antibody (Figure 4F, 4G). A transcriptionally silent population of RNAP-S2p complexes are known to be stably accumulated in splicing speckles [33],[41], which are nuclear domains enriched in splicing machinery, polyA^+^ RNAs, and may be important for post-transcriptional splicing of complex RNAs [42]. The reverse antibody-blocking experiment also confirmed the colocalisation between S2p and S5p sites but produced lower levels of signal depletion (unpublished), as expected due to the larger abundance of S5p sites (Figure 4C). The results from antibody-blocking experiments suggest that most nucleoplasmic S2p sites outside interchromatin clusters also contain the S5p modification, as expected for simultaneous initiation and elongation events on the same gene during cycles of active transcription. Furthermore, S5p-containing structures are in excess of the active factories, demonstrating the presence of discrete sites marked solely by S5p, which represent poised, S5^+^S2p^−^ transcription factories.

### uPA Flanking Genes, CAMK2G and VCL, Are Transcriptionally Active and Associated with Active Transcription Factories Independently of TPA Activation

**Figure 5:**
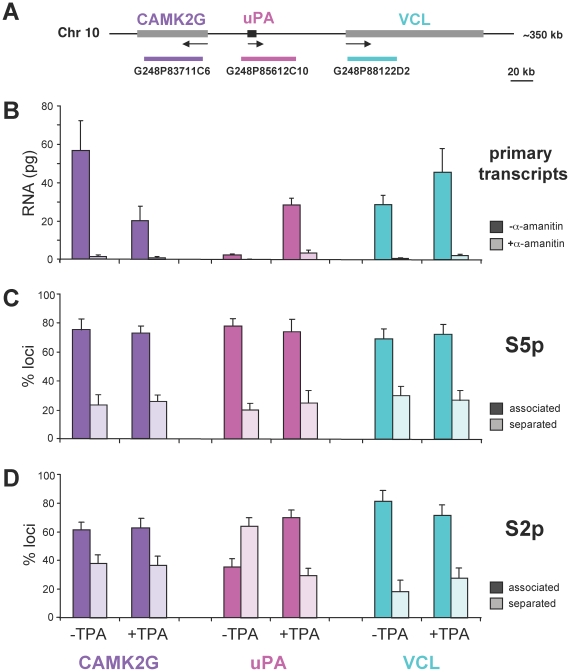
CAMK2G and VCL genes are transcriptionally active and associated with active transcription factories independently of TPA activation. (A) Diagram depicting the uPA locus and the location of the fosmid probes used for the detection of CAMK2G (covering ∼46 kb of genomic sequence), uPA (∼44 kb), and VCL (∼42 kb) genes in cryoFISH experiments. Arrows indicate the 5′-3′ transcription direction. (B) Detection of primary transcripts for the CAMK2G, uPA, and VCL genes in HepG2 cells ±TPA, incubated in the presence or absence of the RNAPII inhibitor α-amanitin. Total RNA was extracted and the levels of primary transcripts determined by qRT-PCR using primers that amplify the exon1-intron1 junctions. Error bars represent the standard deviation of three independent replicates. (C, D) Frequency of association of CAMK2G, uPA, and VCL with RNAP-S5p (C) or -S2p (D) sites before and after TPA treatment for 3 h. The position of each fosmid signal relative to S5p and S2p sites was determined by immuno-cryoFISH, using rhodamine-labelled fosmid probes and antibodies specific for RNAP phosphorylated on S5 or S2 residues (images unpublished). The association of uPA and its flanking (CAMK2G and VCL) genes with RNAP (S5p or S2p) was scored as “associated” (signals overlap by at least 1 pixel) or “separated” (signals do not overlap or are adjacent as in Figure 3B, 3E). The association with S5p sites is similar for all genes across the locus before and after activation (C; *n*_uPA_ = 91 and 93; *n*_CAMK2G_ = 95 and 95; *n*_VCL_ = 90 and 92, for − and +TPA, respectively). CAMK2G and VCL are also associated with S2p sites independently of TPA treatment (D; *n*_CAMK2G_ = 104 and 121; *n*_VCL_ = 79 and 86, for − and +TPA, respectively), whereas the association of the uPA gene with S2p specifically increases upon TPA induction (D; *n*_uPA_ = 254 and 225). The increase in association with S2p factories observed after activation was statistically significant (χ^2^ test, *p*<0.0001).

We have shown large scale repositioning of the uPA locus following TPA treatment of HepG2 cells (Figure 1D). The short genomic separation between uPA gene and neighbouring genes, CAMK2G and VCL (40 kb and 80 kb, respectively; Figure 5A), led us to investigate in more detail how the TPA treatment affected the transcriptional state of the three genes, by comparing the levels of unprocessed transcripts and their association with S5p and S2p factories (Figure 5).

The levels of primary transcripts, produced before and after TPA treatment, were determined by qRT-PCR with primers that amplify the exon1-intron1 junction, using total RNA extracted from HepG2 cells (Figure 5B); cells were treated in parallel with α-amanitin, an inhibitor of RNAP transcription, to discriminate populations of newly made from stable transcripts. Abundant detection of primary transcripts above α-amanitin levels shows that CAMK2G and VCL are actively transcribed prior to TPA activation, whereas uPA primary transcripts are weakly transcribed (Figure 5B; see also Figure 1B, 1C). The levels of CAMK2G and VCL primary transcripts decrease by 2.8-fold and increase by 1.5-fold, respectively, upon TPA treatment, whereas uPA primary transcripts increase by ∼11-fold (Mann-Whitney U test, *p* = 0.05 for the three genes; *n* = 3 independent replicates). Analysis of spliced transcripts of CAMK2G and VCL confirms their active state prior to TPA induction and demonstrates similar effects upon activation (unpublished). Interestingly, low levels of uPA primary transcripts sensitive to α-amanitin treatment are detected prior to activation (Figure 5B), consistent with the detection of uPA protein in a small percentage of HepG2 cells before TPA treatment (Figure 1C). The small and disparate changes in the RNA levels of the two genes flanking uPA are in line with a recent investigation of the *Hoxb* cluster in mouse ES cells, but occur at much shorter genomic distances, in which the *Cbx1* gene, 400 kb downstream of the *Hoxb* cluster, does not change expression levels in spite of increased chromatin repositioning relative to the CT [36]. The behaviour of the uPA flanking genes also agrees with a broader analysis of expression changes across a whole 300 kb region, which undergoes repositioning in response to murine transgenic integration of the β-globin locus-control region, where the expression levels of many genes do not change between the two states [43].

As the levels of primary transcripts at each gene in the locus before and after TPA induction may depend on complex parameters such as the frequency and speed of RNAP elongation, the stability of unprocessed transcripts, and the rate of intron splicing, we investigated whether TPA activation influenced the levels of association of each gene with S5p and S2p sites, using fosmid probes that cover ∼42–46 kb of genomic sequence (Figure 5A). Measurements of the diameters of fosmid and BAC signals yielded average values of 353 nm for the uPA fosmid, in comparison with 586 nm for the BAC probe, which demonstrates a significant improvement in spatial resolution. We find that CAMK2G and VCL are extensively associated with both S5p and S2p sites and to a similar extent, irrespective of TPA treatment (association frequency between 62% and 82%; Figure 5C, 5D). Importantly, the relatively small changes in the levels of primary CAMK2G and VCL transcript upon TPA treatment (Figure 5B) are not reflected by detectable changes in their association with either S5p or S2p sites. This suggests that TPA activation does not influence the extent of CAMK2G and VCL association with the transcription machinery, and thus their state of activity is unlikely to have a major role in the relocation of the uPA locus from its territory.

Similar analyses of uPA gene association with S5p and S2p sites using a fosmid probe (Figure 5C, 5D) confirms the results obtained with the larger BAC probe (Figure 3). Prior to induction, the gene is extensively associated with S5p sites (79%±5%; *n* = 91; Figure 5C), but not with S2p sites (36%±6%; *n* = 254; χ^2^ test, *p*<0.0001; Figure 5D). Upon activation, the uPA fosmid probe associates with S5p and S2p sites to a similar extent (i.e., 75%±9% and 71%±5%, *n* = 93 and 225, respectively; χ^2^ test, *p* = 0.48; Figure 5C, 5D) and at the same levels observed with the BAC probe (∼70%; Figure 3F). Analyses of simulated fosmid signals (Figure S3D, S3E) support the notion that the association of fosmid signals with S5p sites prior to induction, or with both S5p and S2p after activation, are not explained by random processes (χ^2^ test comparisons between experimental and simulated association *p* **_S5p/−TPA_** = 0.0007, *p* **_S5p/+TPA_** = 0.014, *p* **_S2p/+TPA_**<0.0001), whereas the association with S2p sites prior to induction can be (*p* **_S2p/−TPA_** = 0.62).

We were surprised to find that the smaller fosmid probe associates with S2p sites prior to induction to the same extent as the larger BAC probe that also covers the active flanking genes, CAMK2G and VCL (36% and 31%, respectively, χ^2^ test, *p* = 0.26). Simultaneous detection of fosmid and BAC probes in combination with S2p detection (Figure S4B, S4C), confirmed that fosmid and BAC probes associate with S2p sites to a similar extent prior to activation (37%±9% and 28%±9%, respectively; *n* = 46; χ^2^ test, *p* = 0.37; Figure S4C). Unexpectedly, whilst performing these analyses, we observed that a small proportion of uPA loci detected with the fosmid probe (15%±3% and 20%±8%, *n* = 47 and 41, respectively for − and +TPA; χ^2^ test, *p* = 0.57; Figure S4D) were looped out from the signal labelled by the BAC probe independently of TPA activation, in a manner reminiscent of loci looping out from their CTs, but on a much smaller genomic length scale ([4],[5]; Figure 1D). This mechanism provides a rationale for the independent behaviour of neighbouring genes with respect to their association with specific nuclear landmarks, such as shown here for the association with specific RNAP structures.

The fosmid-based analyses allowed us to confirm at higher spatial resolution that the uPA gene is preferentially associated with a subpopulation of RNAP factories, which, prior to induction, contain RNAP-S5p, but not RNAP-S2p. After induction the uPA locus is highly associated with both S5p- and S2p-containing RNAP sites, consistent with its active state.

### TPA Induction of Gene Expression Is Associated with an Increase in the Number of Active uPA Alleles

**Figure 6:**
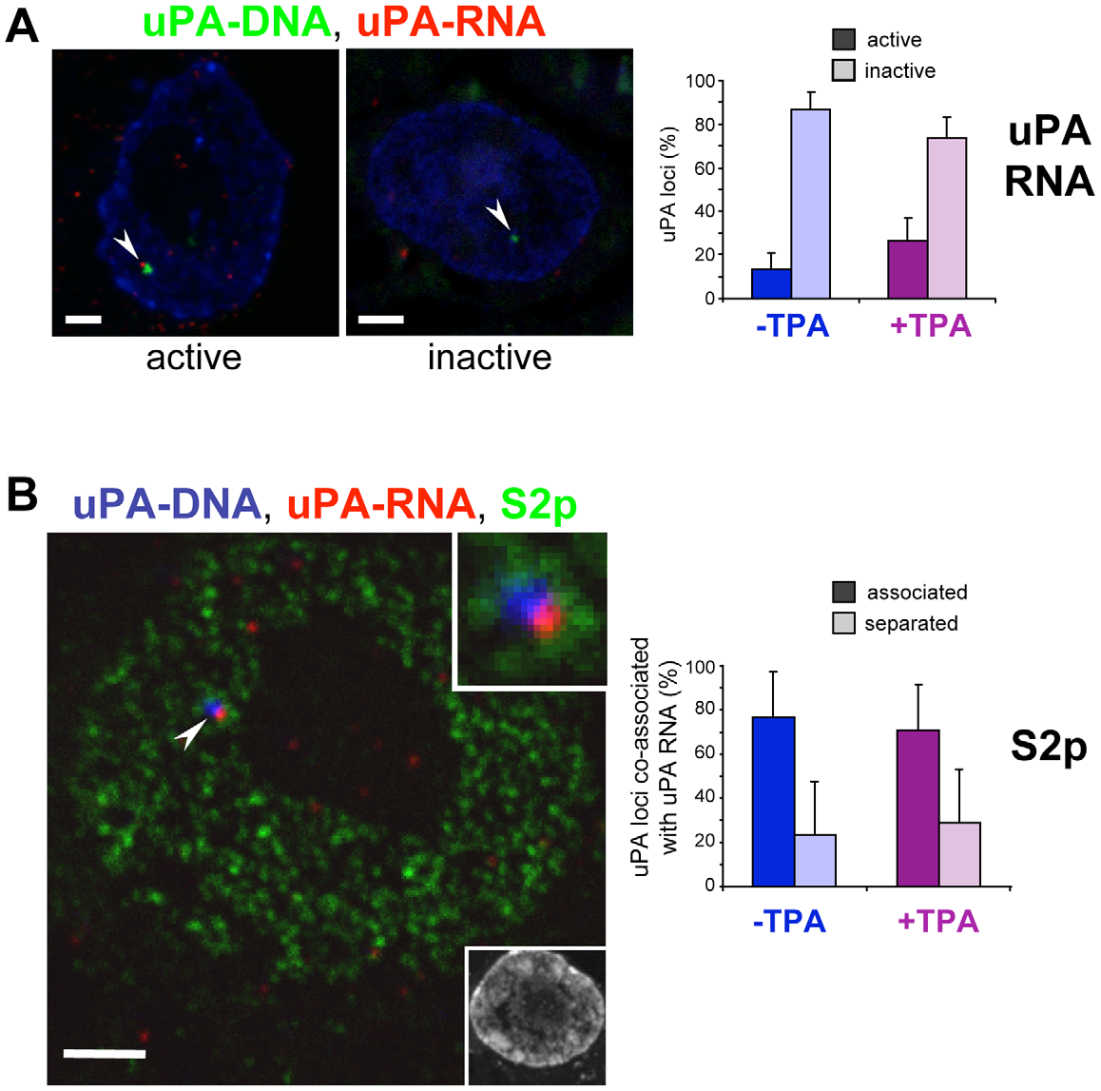
The induction of uPA gene expression is associated with an increase in the number of active alleles. (A) TPA induction of uPA transcripts at the transcription site. uPA gene transcription was detected in HepG2 cells ±TPA by combining RNA and DNA-FISH in the same cryosection. Sections were first hybridized with mixture of five Cy3-labelled fiftymer oligonucleotide probes mapping at introns 4, 5, and 10, and exons 8 and 9 and the signal amplified with fluorescent antibodies. After cross-linking the immunocomplexes detecting uPA RNA, sections were hybridized with the fosmid uPA probe. uPA DNA and RNA signals were scored as “active” (signals overlap or adjacent to each other) or “inactive” (signals do not overlap) to determine the state of activity of the uPA alleles in ±TPA-treated cells. Arrowheads indicate the position of uPA-DNA (green) associated with or separated from uPA-RNA (red) signals. The use of exon probes results in the detection specific cytoplasmic RNA signals. Nucleic acids were counterstained with TOTO-3 (blue). Bars: 2 µm. TPA treatment increases the frequency of uPA allele association with uPA RNA signals from 13% to 27% (*n* = 200 and 216, respectively; *p* = 0.002). (B) The frequency of co-association of the uPA gene (blue) with RNAP-S2p (green) and uPA-RNA (red) was determined by triple labelling, using the fosmid uPA probe, antibodies specific for RNAP phosphorylated at S2 residues (H5), and Cy3-labelled oligonucleotide probes. The frequency of association of active uPA alleles with S2p sites was scored as “associated” (signals overlap by at least 1 pixel) or “separated” (signals do not overlap and adjacent signals). Arrowhead indicates the position of uPA-DNA (blue) and uPA-RNA (red) relative to S2p (green). Nucleic acids were counterstained with TOTO-3 (inset). Bar: 2 µm. Most uPA genes that are actively transcribed are also associated with RNAP-S2p, confirming that this RNAP modification marks active factories.

In order to investigate whether the extent of uPA gene association with active transcription factories reflects their transcriptional activity, we combined the detection of the uPA locus by DNA-FISH with the visualisation of uPA transcripts by RNA-FISH using five tagged oligoprobes mapping at introns 4, 5, and 10, and exons 8 and 9. We find that the locus is already transcriptionally active prior to activation, with 13%±8% of uPA alleles showing an association with RNA-FISH signals (*n* = 200 loci; Figure 6A), consistent with the detection of uPA protein and transcripts before induction (Figures 1C and 5B, respectively). RNase control experiments confirmed the specificity of the discrete RNA-FISH signals observed within the nucleus (Figure S5). We also show that the frequency of active alleles increases 2-fold, to 27%±10%, after TPA treatment (*n* = 216; χ^2^ test comparison for − and +TPA, *p* = 0.0022; Figure 6A), in agreement with the 2-fold increase observed in the extent of uPA association with RNAP-S2p (Figure 4B). The extent of uPA gene association with RNA signals after activation (27%) is consistently smaller than its association with S2p sites (70%; Figure 5D). However, it must be considered that the efficiency of detection of newly made transcripts at the site of transcription depends on the abundance of RNAP loading at each single gene, the stability of newly made transcripts at the site of synthesis, and the rate of splicing, and therefore is likely to provide a lower estimate for the frequency of gene activity. Intron lengths at the uPA gene are at most ∼900 bp, and small introns can be promptly removed within seconds of synthesis. In contrast, the level of uPA gene association with S2p sites can provide a higher estimate, with the caveat that these levels of association may also reflect, in part, an indirect colocalisation of uPA loci with transcription factories associated with the flanking genes.

To verify whether detection of newly synthesized uPA transcripts at the uPA locus occurred concomitantly with its association with active, S2p factories, we performed triple labelling experiments in which we simultaneously detected the uPA locus, uPA transcripts, and S2p active factories (Figure 6B). We found that most uPA loci associated with an RNA signal are also associated with S2p sites both before and after TPA treatment (76% and 71%, *n* = 70 and 75, respectively; χ^2^ test, *p* = 0.80; Figure 6B), confirming that S2p sites are active sites of transcription. As expected from the higher levels of uPA gene association with S2p than RNA-FISH sites, we find that of all the uPA loci associated with S2p sites (∼70%) only half are also associated with uPA transcripts (unpublished; see also [36]). This difference is likely to reflect technical limitations in the detection of transcripts of short genes containing only small introns.

Our analyses of uPA gene association with different phosphorylated forms of RNAP and with newly made transcripts show that the vast majority of uPA alleles are associated with poised S5p^+^S2p^−^ transcription factories prior to activation. We also identify a small population of alleles transcribed at low levels prior to activation and predominantly associated with sites that are marked by S2p (Figures 5B and 6B). Upon activation, uPA alleles become associated with RNAP sites marked by both S2p and S5p. We consistently find a 2-fold increase in the association of the uPA gene association with S2p sites or uPA transcripts (Figures 3C, 3F, 5D, and 6A), identifying an increased frequency of transcription upon TPA induction.

### uPA Gene Association with Poised or Active Factories Occurs Irrespectively of the Locus Position Relative to Its CT

**Figure 7:**
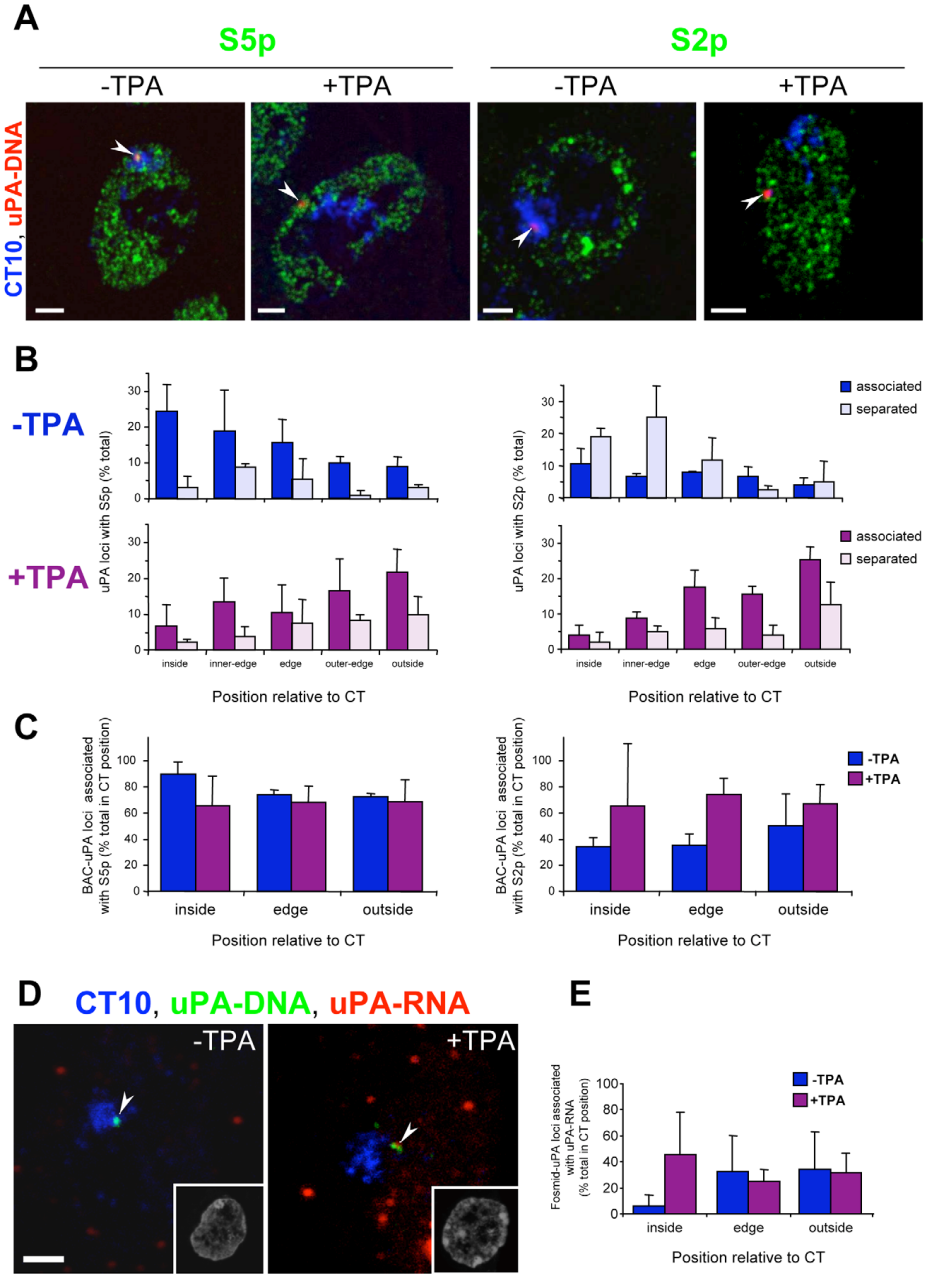
Increased association of uPA locus with active transcription factories upon TPA activation is independent of the large-scale repositioning of the uPA locus relative to its chromosome territory. (A) The position of the uPA locus (uPA-DNA, red) relative to chromosome 10 (CT10, blue) and to S5p and S2p sites (green) was determined in HepG2 cells ±TPA activation for 3 h, by immuno-cryoFISH using a digoxigenin-labelled BAC probe containing the uPA locus, whole chromosome 10 paint, and antibodies specific for RNAP phosphorylated on S5 or S2 residues. Arrowheads indicate the position of uPA loci. Nuclei acids were counterstained with DAPI (images unpublished). Bars: 2 µm. (B) The frequencies of uPA loci which are associated or separated from RNAP-S5p (left hand column) or RNAP-S2p (right hand column) were measured at each position relative to the CT (“inside,” “inner-edge,” “edge,” “outer-edge,” and “outside”) before and after TPA induction. uPA locus association with S5p and S2p sites was detected in all position analyzed. (C) The proportion of uPA loci that associate with RNAP-S5p (left hand column) or RNAP-S2p (right hand column) before and after TPA activation was determined at each CT position. Association of the locus with S5p or S2p sites before and after activation are independent of its position relative to the CT. (D) Combined detection of the chromosome 10 (CT10, blue), the uPA gene (uPA-DNA, green), and uPA RNA (uPA-RNA, red) was performed by RNA- and DNA-FISH using a whole chromosome paint, the uPA fosmid probe, and Cy3-conjugated oligonucleotide probes. Nuclei acids were counterstained with TOTO-3 (insets). Arrowheads indicate the position of uPA-RNA and uPA-DNA signals. Bar: 2 µm. (E) The proportion of uPA genes that associate with uPA RNA before and after TPA activation was calculated at each CT position. Prior to activation, uPA genes at the CT interior are less frequently transcribed than loci outside the territory. After TPA treatment, the frequency of transcriptional events is the same at all CT positions.

We next investigated whether the increased frequency of uPA gene transcription or association with transcription factories were dependent on the locus position relative to its CT, both before and after activation, when the locus is preferentially located at the CT interior and exterior, respectively. We performed triple labelling cryoFISH experiments for chromosome 10, the uPA locus, and transcription factories, before and after activation (Figure 7A–C). Analyses were initially performed with BAC probes, but also confirmed with fosmid probes (Figure S6). As previously observed in double labelling experiments (Figure 1D), the uPA locus was preferentially located at the CT interior and associated with S5p transcription factories before activation (Figure 7B). TPA activation induced the relocation of most uPA loci to the CT exterior and an association with factories marked with both S5p and S2p (Figure 7B).

To determine whether the association of the uPA locus with poised or active factories was dependent on its position relative to the CT, we calculated the proportion of uPA locus association with RNAP at each CT position (Figure 7C). For simplicity, the data for the three regions around the edge of the CT (“inner-edge,” “edge,” and “outer-edge”) were pooled into a single region, but analyses of the five regions gave similar results. Surprisingly, we found that the association of the uPA locus with S5p occurred with similar frequency at all locations relative to the CT, independently of TPA activation (*n* = 90 and 134 loci, respectively; logistic regression analysis, *p* = 0.20; Figure 7C). This shows that the CT interior is accessible to the transcription machinery and does not preclude the interaction of a primed gene with poised, S5p^+^S2p^−^ factories. In the case of S2p, we also found that the uPA locus associates with active transcription factories with similar frequencies across the different CT regions before and after TPA activation (*n* = 151 and 104, respectively; logistic regression analysis, *p* = 0.10). The increase in association of uPA loci with active factories marked by S2p after TPA is statistically significant (*p*<0.0001), but the effect of TPA on the level of association is the same across all positions relative to the CT (*p* = 0.62; Figure 7C). These results show that the uPA gene associates with poised or active transcription factories with similar frequencies across the different CT regions both before and after transcriptional activation. Therefore, looping of the uPA locus out of its CT is not required for the association of the uPA gene with active transcription factories.

To investigate whether the large-scale chromatin movements that accompany TPA induction of the uPA gene had an effect on the association of the flanking genes, CAMK2G and VCL, with active (S2p) factories, we performed triple labelling cryoFISH experiments for chromosome 10, the CAMK2G, or VCL loci detected with fosmid probes and active (S2p) factories (Figure S7). We find that the association of CAMK2G or VCL with S2p also occurs with similar frequency at all locations relative to the CT, both before and after TPA activation (logistic regression analysis, *p* = 0.84 and 0.64 for CAMK2G and VCL, respectively; *n* **_CAMK2G/−TPA_** = 108, *n* **_CAMK2G/+TPA_** = 128, *n* **_VCL/−TPA_** = 147, *n* **_VCL/+TPA_** = 134). These results show that the association of the uPA, CAMK2G, and VCL genes with RNAP-S2p occurs independently of CT position. A recent analysis of gene activation induced by the insertion of a strong (ß-globin) enhancer in a gene rich-region also showed no effect on the frequency of locus association with active transcription factories at different positions relative to the CT [43], although this region preferentially localises at the CT edge. In the case of the murine *Hoxb* locus, a small preferential association of *Hoxb1* and flanking genes with active transcription factories is observed outside the CT upon retinoic acid treatment [36]. Different mechanisms of gene regulation may act on different genes and depend on the kinetics of induction over the shorter activation (3 h) of the uPA gene by TPA treatment in comparison with retinoic acid treatment for several days to induce *Hox* genes.

Finally, to investigate whether the CT position of the uPA gene has an influence on its transcriptional activity, we labelled the uPA gene, chromosome 10, and uPA transcripts simultaneously (Figure 7D, 7E). We used the fosmid uPA probe for highest spatial resolution. We find that the uPA gene is transcribed with the same frequency irrespectively of its CT position upon TPA activation (logistic regression analysis, *p* = 0.74; *n* = 100). These results differ from the murine *Igf2bp1* and *Cbx1* genes, flanking the *Hoxb* cluster, which are also transcriptionally active at all CT positions, independently of *Hoxb* induction, but are preferentially active outside the CT [36]. Difficulties in the detection of the *Hoxb1* transcripts did not allow a similar analysis of allelic transcription upon induction [36], to help establish how general the correlation is between gene positioning outside the CT and transcriptional states.

Prior to activation, we unexpectedly found that the largest fraction of uPA loci, which are internal to the CT, are less likely to be transcriptionally active (logistic regression analysis, *p* = 0.0001; *n* = 103), whereas the smaller proportion of uPA loci not located at the preferred internal CT position is transcribed at the same frequency as upon TPA induction (Figure 7E). These results suggest that the internal CT positioning has a silencing effect on the primed uPA locus prior to its induction, which helps prevent transcript elongation or interferes with transcript stability, revealing unexpected properties of locus positioning within the nuclear landscape.

In summary, our analyses of the uPA gene prior to induction showed that it was (a) preferentially positioned at the interior of its CT; (b) in a poised state, characterized by open chromatin configuration and the presence of RNAP-S5p at regulatory regions; and (c) preferentially associated with poised, S5p^+^S2p^−^ transcription factories. Transcriptional activation induces large-scale relocation of the gene towards the CT exterior and a preferred association with factories containing both (S5p and S2p) RNAP modifications, as expected in the active state. Although the correlation between looping out of the CT and the change in RNAP configuration suggested that the external position might favour transcriptional activation, triple-labelling experiments showed that the position of the uPA locus relative to its CT and the association with poised or active transcription factories are independent events. RNA-FISH experiments confirm that after TPA induction both external and internal positions of the uPA gene, with respect to its CT, are equally competent for transcription. However, positioning of the uPA locus inside the CT, before activation, may help control the levels of transcription, as uPA genes that are found outside of the CT before TPA treatment are more likely to be transcribed (Figure 7E).

Our findings reinforce the idea that the interior of CTs is not repressive for the association of genes with transcription machinery, suggesting that large-scale chromatin movements are unlikely to be necessary for genes to find transcription factories, although they may influence the extent of association for specific subsets of genes. This study expands current models of gene regulation by showing that silent genes can be associated with poised transcription factories and that factory association and gene position relative to the CT can be independent factors. Our results are also compatible with the notion that poised transcription factories represent a sub-population of specialized sites that may allow primed genes to respond rapidly and efficiently to specific activation signals.

## Materials and Methods

A detailed description of the experimental procedures is given in Text S1.

### Cell Culture, RNA Detection, and Western Blotting

HepG2 cells were cultured in the absence or presence of 100 ng/ml TPA (Sigma) for the indicated times as previously described [27]. Treatment of HepG2 cells with 1 µM flavopiridol (1 h; Sanofi-Aventis) was used for the inhibition of RNAP-S2p phosphorylation by CDK9, and 75 µg/ml α-amanitin (5 h; Sigma) to inhibit RNAP transcription. For the quantification of mature and unprocessed transcript levels of uPA, CAMK2G, or VCL genes, total RNA was extracted and amplified by RT-PCR. Western blotting was performed using total HepG2 protein extracts and antibodies specific to different RNAP phosphoforms. Experimental details and information about the antibodies used can be found in Text S1.

### MN-ChIP and PCR Reactions

Chromatin cross-linking, MNase digestion, and immunoprecipitation were performed as described previously [31]. See Text S1 for primer sequences (Table S1), antibodies used, and experimental details.

### Immunofluorescence, Ultracryosectioning, and cryoFISH

uPA protein expression was detected with specific rabbit antiserum antibodies. For high-resolution imaging using cryoFISH, ultrathin cryosections (∼140–150 nm thick) were immunolabelled and/or labelled by fluorescence in situ hybridization (FISH) essentially as described before [14]. RNA-FISH was performed using oligonucleotide probes (http://www.singerlab.org/protocols). See Text S1 for information about the antibodies and probes used, and for experimental details.

### Microscopy, Quantitative Image Analyses, and Statistics

Images were acquired by confocal microscopy and analysed quantitatively. Statistical analyses were performed using χ^2^ test, logistic regression analysis, ANOVA, Student *t*-test, or Mann-Whitney U test. See Text S1 for further details.

## Supporting Information

Figure S1 **H3K9me2 histone modification is present at H19 gene promoter, but not the uPA gene promoter.** Cross-linked, sonicated chromatin from ±TPA-treated HepG2 cells was digested with MN for 50 min before immunoprecipitation with antibodies that recognize lysine 9 dimethylated histone H3 (H3K9me2), associated with closed chromatin. Control (“unrelated”) antibodies were polyclonal anti-uPAR antibodies. Immunoprecipitated DNA was amplified using primers spanning the 5′ portion of the imprinted H19 gene and the uP fragment of the uPA gene (see scheme in Figure 2D).
(0.72 MB TIF)

Figure S2 **Characterization of antibodies against different phosphorylated forms of RNAP.** (A, B) Reactivity of different RNAP antibodies against hyper- (II_O_) and hypophosphorylated (II_A_) forms of the largest subunit of RNAP (RPB1) was assessed by Western blotting using total protein extracts from HepG2 cells treated for 1 h in the absence (A) or presence (B) of 1 µM flavopiridol, a specific inhibitor of CDK9, the Ser2 kinase. Both II_O_ and II_A_ bands are detected by antibody N-20 (A), raised against the amino-terminus of RPB1, which binds independently of phosphorylation. Antibodies against S5p (4H8 and H14) or S2p (H5) only detect the II_O_ band (A, B). Treatment of Western blots with alkaline phosphatase (AP; A) prior to immunolabelling reveals the specificity of 4H8, H14, and H5 antibodies for phosphorylated epitopes, and has no effect on the binding of an antibody to the N terminus of RPB1. The specificity of H5 antibodies to the S2p modification is shown by loss of binding in flavopiridol-treated samples (B). Binding of 4H8 and H14 antibodies to II_O_ band is insensitive to flavopiridol treatment in these conditions, consistent with their specificity for the Ser5 modification (S5p) catalyzed by CDK7, as previously shown (B and [21]). Protein loading was controlled using histone H2B antibodies. (C–F) Cryosections (∼150 nm thick) from HepG2 cells were treated ± AP prior to immunolabelling with phosphorylation dependent RNAP antibodies. Sections were indirectly immunolabelled with antibodies against RNAP-S5p (4H8; C, E), or RNAP-S2p (H5; D, F). Absence of signal after pre-treatment of cryosections with AP (E, F) shows that 4H8 and H5 antibodies bind specifically to phosphorylated epitopes, and do not detect unphosphorylated RPB1. Nucleic acids were counterstained with TOTO-3 (insets). Bar: 2 µm.
(8.10 MB TIF)

Figure S3 **Frequency of association of simulated uPA loci with RNAP-S5p and RNAP-S2p sites.** (A) Diagram of the genomic location of the uPA gene and the regions covered by the BAC (RP11-417O11; ∼228 kb) and fosmid (G248P85612C10; ∼44 kb) probes used for FISH experiments. Arrows indicate the 5′-3′ transcription direction. (B, D) To analyse the frequency of association of a simulated uPA locus positioned at random coordinates with RNAP-S5p or -S2p sites, we generated a new image containing the original experimental S5p (B, D; green) or S2p (images unpublished) distribution, and the experimental uPA signal (Exp-uPA; blue; arrowheads), and an additional, simulated uPA signal with the same number of pixels, but positioned at random nucleopasmic coordinates (Siml-uPA; red; arrows). This analysis was performed for both BAC (B) and fosmid (D) experiments presented in Figures 3B, 3C, 3E, 3F and 5C, 5D, respectively. Nucleic acids were counterstained with TOTO-3 (insets). Bars: 2 µm. (C, E) Frequency of association of experimental and simulated uPA loci with RNAP-S5p and RNAP-S2p in the same experimental images of HepG2 cells treated ±TPA. Experimental uPA loci associate more frequently with S5p sites than simulated loci, positioned at random nucleoplasmic coordinates, both before and after TPA treatment, for both BAC (C) and fosmid (D) probes. In contrast, the level of association of experimental BAC or fosmid loci with S2p sites is similar to the levels of simulated (random) loci before, but not after, TPA activation. This confirms that the increased association of the uPA gene with S2p sites detected following activation is not due to random processes and is not affected by the size of the probe used. The numbers of simulated sites were *n*_BAC,S5p_ = 68 and 62; *n*_BAC,S2p_ = 69 and 75; *n*_fosmid,S5p_ = 47 and 40; *n*_fosmid,S2p_ = 50 and 46, for − and +TPA, respectively.
(8.58 MB TIF)

Figure S4 **The uPA gene loops out of its chromatin domain.** (A) Diagram illustrating the genomic location of the uPA gene and the regions covered by the BAC (RP11-417O11; ∼228 kb, blue) and fosmid (G248P85612C10; ∼44 kb, red) probes used for FISH experiments. Arrows indicate the 5′-3′ transcription direction. (B) The association of BAC and fosmid signals (arrowhead) relative to RNAP-S2p sites (green) was determined simultaneously by immuno-cryoFISH before (unpublished image) and after (B) TPA activation for 3 h, using digoxigenin-labelled BAC (blue) and rhodamine-labelled fosmid (red) probes. High magnification images show examples of the co-association of both BAC- and fosmid-uPA signals with S2p sites (top) or the association of fosmid-uPA signal, but not the BAC signal with S2p sites (bottom). Nucleic acids were counterstained with DAPI (inset). Bar: 2 µm. (C) Frequency of the association of BAC or fosmid signals with S2p sites is similar between probes (χ^2^ test, *p* = 0.37 and *p* = 0.81, *n* = 46 and 40, for − and +TPA, respectively). Error bars are standard deviations from two replicate experiments. (D) Fosmid signals (red) can loop out of BAC foci (green). Arrowheads indicate the position of BAC and fosmid signals. Insets show higher magnification images. Nucleic acids were counterstained with TOTO-3 (blue). Bar: 2 µm. Frequency of co-localisation of BAC foci with fosmid-uPA signals show that 15%–20% of uPA alleles detected with the fosmid probe loop out from the BAC signals. The difference between the levels of fosmid looping ±TPA was not statistically significant (χ^2^ test, *p* = 0.57; *n* = 47 and 41, for − and +TPA, respectively).
(6.78 MB TIF)

Figure S5 **Control experiments for cryo-RNA-FISH.** (A–D) Cryosections (∼150 nm thick) of HepG2 cells were hybridised with Cyanine3 labelled uPA oligonucleotide probes before (A) and after (B–D) TPA activation. Inspection of nucleoplasmic regions identifies frequent uPA-RNA signals in TPA-treated cells (B, arrowheads). Pre-incubation of sections with RNase A (C) or omission of oligonucleotide probes (D) abolishes most uPA-RNA signals within the nucleoplasm, demonstrating its specificity. Nucleic acids were counterstained with TOTO-3 (red). Bars: 2 µm.
(8.38 MB TIF)

Figure S6 **Detection of the uPA gene with a fosmid probe recapitulates the CT looping and position-independent association with S5p and S2p factories observed with the BAC probe.** (A) The position of the uPA locus (fosmid-uPA, green) relative to the chromosome 10 territory (CT10, red) was determined in HepG2 cells, before and after TPA activation for 3 h, by cryoFISH using a whole chromosome 10 paint and a digoxigenin-labelled uPA fosmid probe. The positions of uPA loci were scored as “inside,” “inner-edge,” “edge,” “outer-edge,” and “outside” relative to its CT as in Figure 1D. Nucleic acids were counterstained with TOTO-3 (blue). Arrowheads indicate uPA loci. Bar: 2 µm. The histogram shows that the fosmid-uPA probe recapitulates the TPA-induced CT looping observed with the BAC probe (Figure 1D), as expected. In the inactive state, the locus is preferentially localized at the CT interior (61% loci inside or at the inner-edge, *n* = 234 loci), and relocates to the exterior upon activation (58% loci at outer-edge or outside, *n* = 230 loci; χ^2^ test, *p*<0.0001). (B) The proportion of uPA loci detected using the fosmid probe, which associate with RNAP-S5p before and after TPA activation, was calculated at each CT position (inside, edge, outside) as for the BAC probe (Figure 7C). Association of the locus with S5p sites before and after activation is independent of its position relative to the CT (logistic regression analysis; *p* = 0.18 and *p* = 0.26 before and after TPA, *n* = 68 and 71, respectively). Overall no effect of TPA treatment on the association of the uPA gene with S5p was detected (logistic regression analysis, *p* = 0.54). Association of uPA loci detected with the fosmid probe with S2p sites before and after activation is also independent of its position relative to the CT (logistic regression analysis, *p* = 0.27 and *p* = 0.79 before and after TPA, *n* = 113 and 140, respectively). This same analysis also detected an increased association of uPA gene with S2p sites upon activation (logistic regression analysis, *p* = 0.0004).
(5.63 MB TIF)

Figure S7 **uPA-flanking genes, CAMK2G and VCL, associate with S2p factories independently of CT position or TPA activation.** The association of CAMK2G or VCL loci with RNAP-S2p were determined relative to the chromosome 10 territory (CT10) in HepG2 cells, before and after TPA activation (3 h), by cryoFISH using a whole chromosome 10 paint and digoxigenin-labelled CAMK2G and VCL fosmid probes. (A) Images represent examples of CAMK2G loci (red, arrowheads) that co-localise with RNAP-S2p (green) inside (left) or outside (right) of CT10 (blue). Nucleic acids were counterstained with DAPI (insets). Bar: 2 µm. (B) The proportion of CAMK2G or VCL loci, which associate with RNAP-S2p, was determined at each CT position (inside, edge, outside), before and after TPA activation. Association of either gene with S2p is independent of their position relative to the CT and to TPA treatment.
(5.98 MB TIF)

Figure S8 **Examples of classification criteria for the association of uPA loci with RNAP-S5p and RNAP-S2p sites.** The position of the uPA locus (red) with S5p and S2p sites (green) was determined by immuno-cryoFISH using a rhodamine-labelled BAC probe containing the uPA locus and antibodies specific for RNAP phosphorylated at residues S5 or S2 of the CTD. Associated uPA loci co-localise with S5p or S2p sites if signals overlap by at least a single pixel, whereas separated sites do not show overlap of the two signals and include loci that may touch an RNAP site without signal overlap.
(4.11 MB TIF)

Table S1 **MN-ChIP primers. List of primers used in MN-ChIP analyses in 5′ to 3′ orientation.**
(0.06 MB DOC)

Text S1 **Supplementary information.**
(0.10 MB DOC)

## Abbreviations

CAMK2G: calcium calmodulin-dependent protein kinase II gamma
CT: chromosome territory
CTD: carboxy-terminal domain
FISH: fluorescence in situ hybridization
MN: micrococcal nuclease
MN-ChIP: MN-coupled Chromatin ImmunoPrecipitation
RNAP: RNA Polymerase II
TPA: tetradecanoyl phorbol acetate
uPA: urokinase-type Plasminogen Activator
VCL: vinculin
