# Supplementary figures and images for: Poised Transcription Factories Prime Silent uPA Gene Prior to Activation
Source: PLoS Biol. 2010 Jan 5;8(1):e1000270. doi: 10.1371/journal.pbio.1000270 (PMC2797137; doi:10.1371/journal.pbio.1000270)

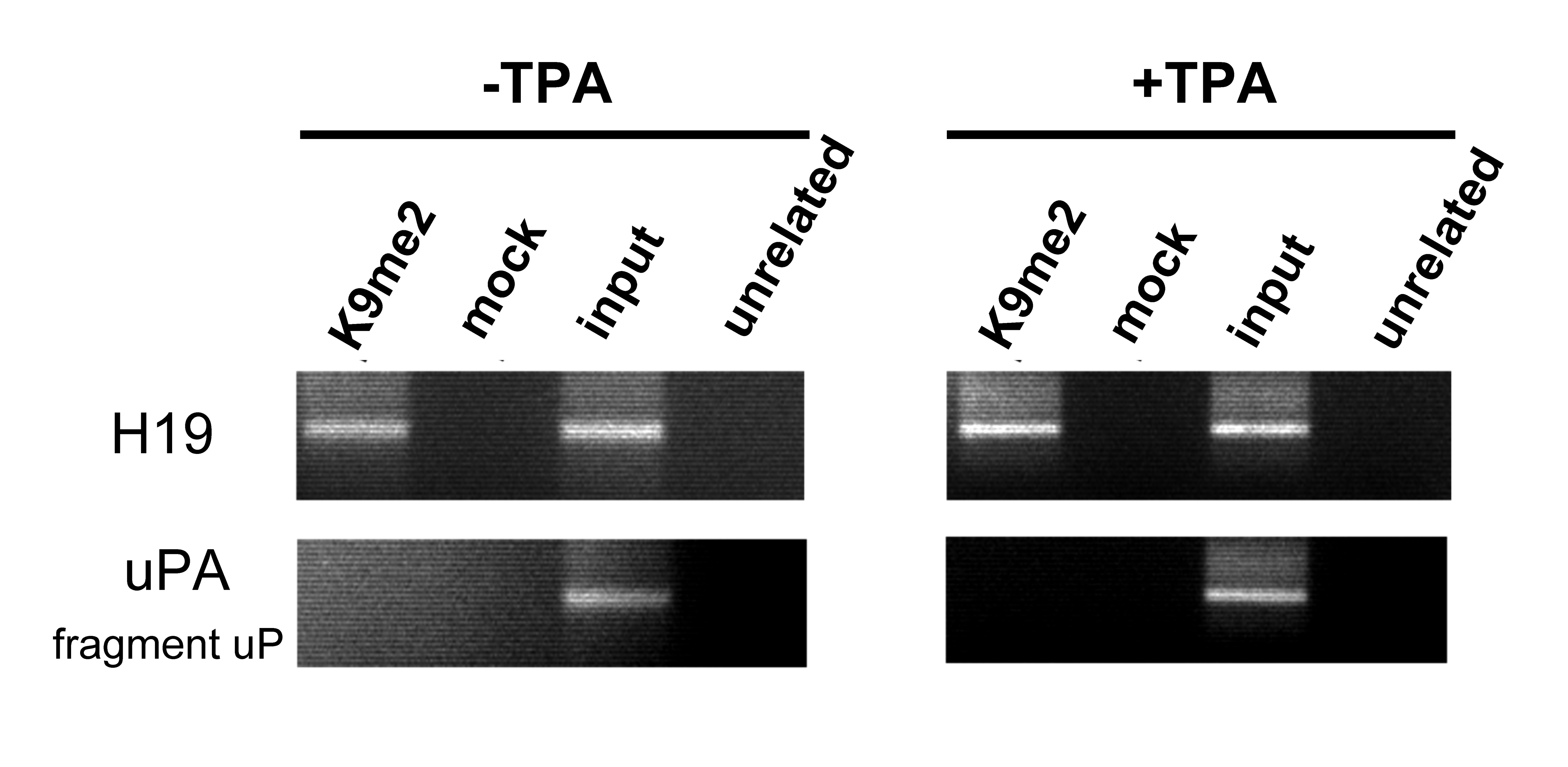

Supplement: Figure S1 — H3K9me2 histone modification is present at H19 gene promoter, but not the uPA gene promoter. Cross-linked, sonicated chromatin from ±TPA-treated HepG2 cells was digested with MN for 50 min before immunoprecipitation with antibodies that recognize lysine 9 dimethylated histone H3 (H3K9me2), associated with closed chromatin. Control (“unrelated”) antibodies were polyclonal anti-uPAR antibodies. Immunoprecipitated DNA was amplified using primers spanning the 5′ portion of the imprinted H19 gene and the uP fragment of the uPA gene (see scheme in Figure 2D). (0.72 MB TIF) [file pbio.1000270.s001.tif]

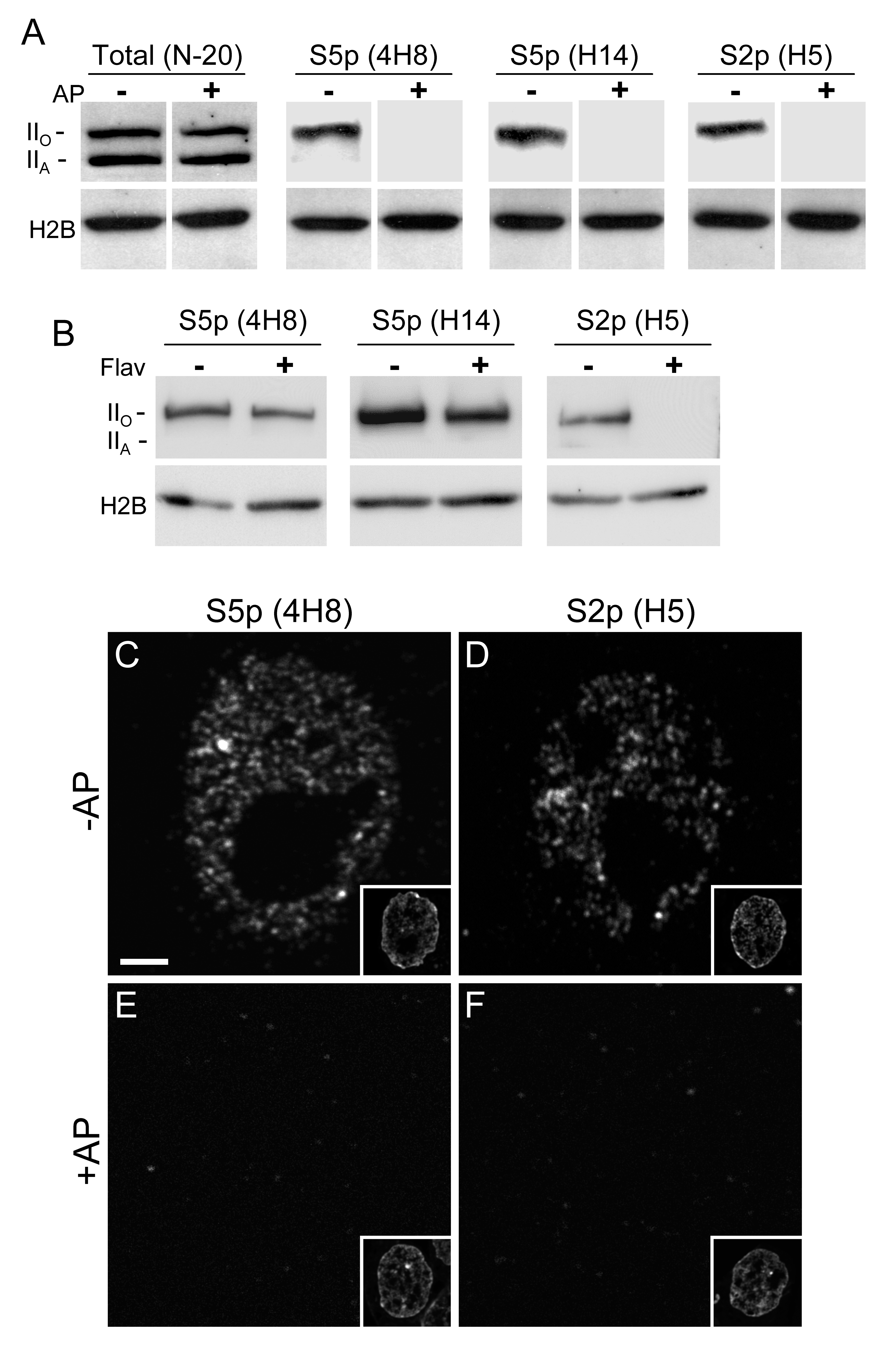

Supplement: Figure S2 — Characterization of antibodies against different phosphorylated forms of RNAP. (A, B) Reactivity of different RNAP antibodies against hyper- (IIO) and hypophosphorylated (IIA) forms of the largest subunit of RNAP (RPB1) was assessed by Western blotting using total protein extracts from HepG2 cells treated for 1 h in the absence (A) or presence (B) of 1 µM flavopiridol, a specific inhibitor of CDK9, the Ser2 kinase. Both IIO and IIA bands are detected by antibody N-20 (A), raised against the amino-terminus of RPB1, which binds independently of phosphorylation. Antibodies against S5p (4H8 and H14) or S2p (H5) only detect the IIO band (A, B). Treatment of Western blots with alkaline phosphatase (AP; A) prior to immunolabelling reveals the specificity of 4H8, H14, and H5 antibodies for phosphorylated epitopes, and has no effect on the binding of an antibody to the N terminus of RPB1. The specificity of H5 antibodies to the S2p modification is shown by loss of binding in flavopiridol-treated samples (B). Binding of 4H8 and H14 antibodies to IIO band is insensitive to flavopiridol treatment in these conditions, consistent with their specificity for the Ser5 modification (S5p) catalyzed by CDK7, as previously shown (B and [21]). Protein loading was controlled using histone H2B antibodies. (C–F) Cryosections (∼150 nm thick) from HepG2 cells were treated ± AP prior to immunolabelling with phosphorylation dependent RNAP antibodies. Sections were indirectly immunolabelled with antibodies against RNAP-S5p (4H8; C, E), or RNAP-S2p (H5; D, F). Absence of signal after pre-treatment of cryosections with AP (E, F) shows that 4H8 and H5 antibodies bind specifically to phosphorylated epitopes, and do not detect unphosphorylated RPB1. Nucleic acids were counterstained with TOTO-3 (insets). Bar: 2 µm. (8.10 MB TIF) [file pbio.1000270.s002.tif]

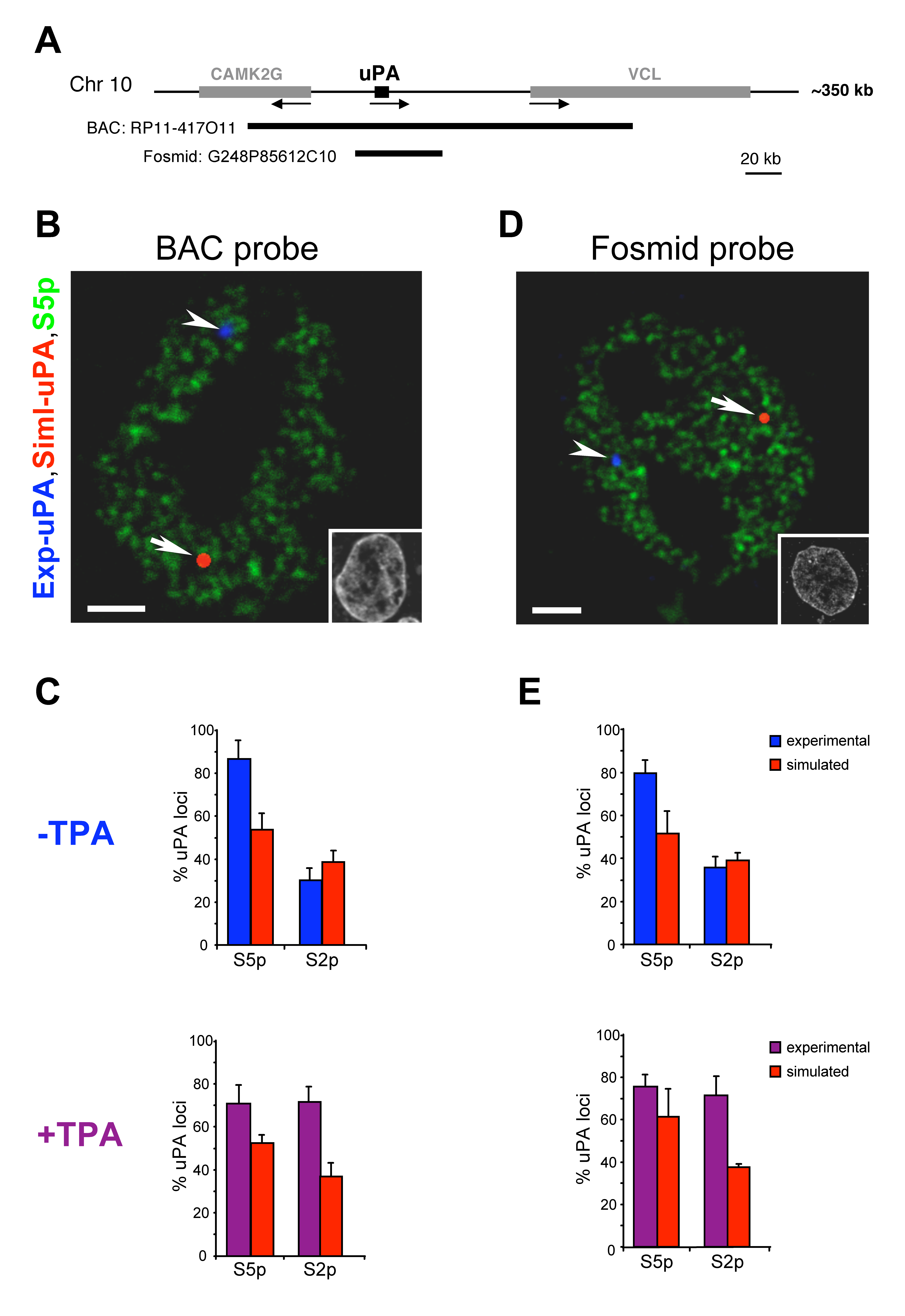

Supplement: Figure S3 — Frequency of association of simulated uPA loci with RNAP-S5p and RNAP-S2p sites. (A) Diagram of the genomic location of the uPA gene and the regions covered by the BAC (RP11-417O11; ∼228 kb) and fosmid (G248P85612C10; ∼44 kb) probes used for FISH experiments. Arrows indicate the 5′-3′ transcription direction. (B, D) To analyse the frequency of association of a simulated uPA locus positioned at random coordinates with RNAP-S5p or -S2p sites, we generated a new image containing the original experimental S5p (B, D; green) or S2p (images unpublished) distribution, and the experimental uPA signal (Exp-uPA; blue; arrowheads), and an additional, simulated uPA signal with the same number of pixels, but positioned at random nucleopasmic coordinates (Siml-uPA; red; arrows). This analysis was performed for both BAC (B) and fosmid (D) experiments presented in Figures 3B, 3C, 3E, 3F and 5C, 5D, respectively. Nucleic acids were counterstained with TOTO-3 (insets). Bars: 2 µm. (C, E) Frequency of association of experimental and simulated uPA loci with RNAP-S5p and RNAP-S2p in the same experimental images of HepG2 cells treated ±TPA. Experimental uPA loci associate more frequently with S5p sites than simulated loci, positioned at random nucleoplasmic coordinates, both before and after TPA treatment, for both BAC (C) and fosmid (D) probes. In contrast, the level of association of experimental BAC or fosmid loci with S2p sites is similar to the levels of simulated (random) loci before, but not after, TPA activation. This confirms that the increased association of the uPA gene with S2p sites detected following activation is not due to random processes and is not affected by the size of the probe used. The numbers of simulated sites were n BAC,S5p = 68 and 62; n BAC,S2p = 69 and 75; n fosmid,S5p = 47 and 40; n fosmid,S2p = 50 and 46, for − and +TPA, respectively. (8.58 MB TIF) [file pbio.1000270.s003.tif]

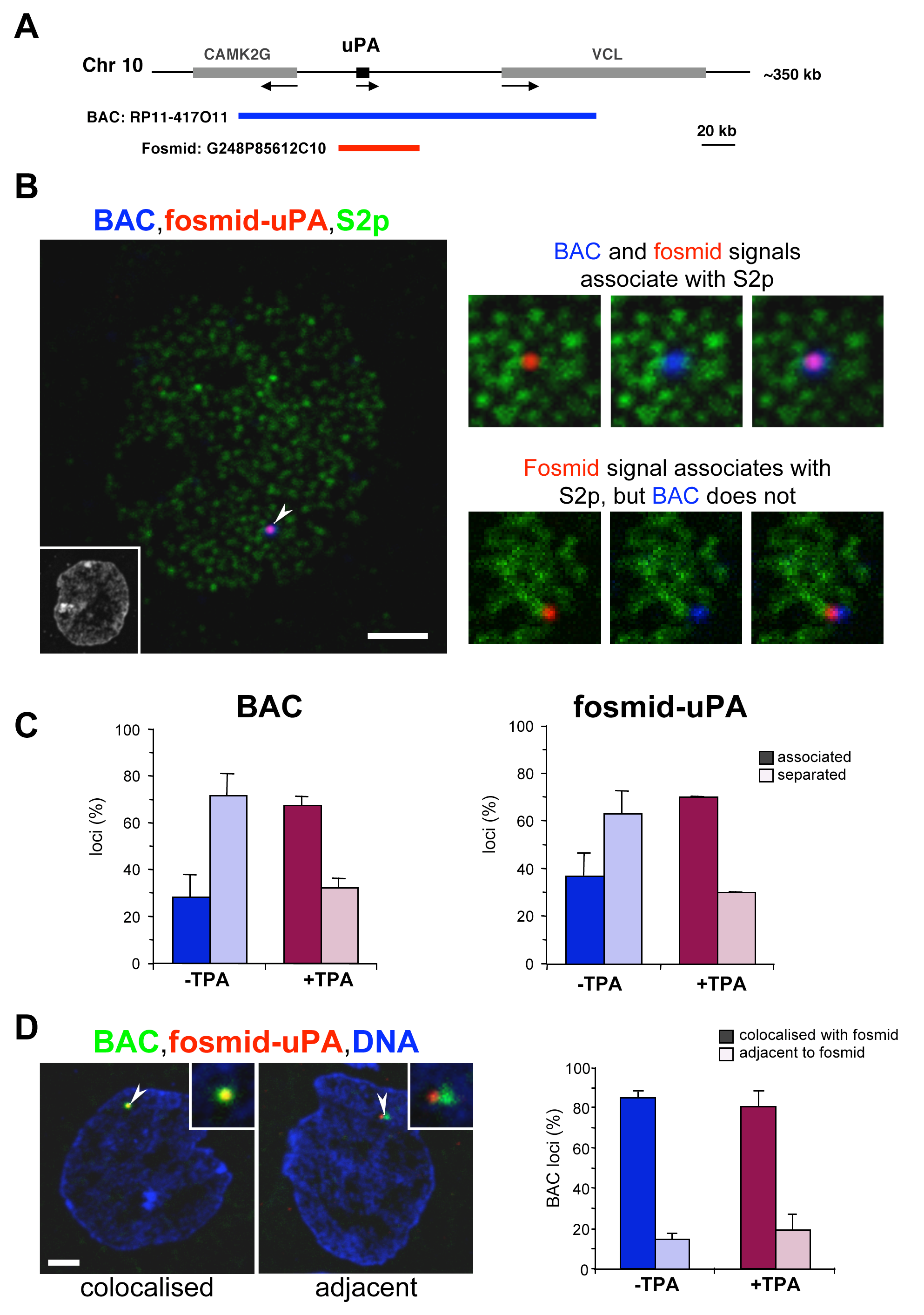

Supplement: Figure S4 — The uPA gene loops out of its chromatin domain. (A) Diagram illustrating the genomic location of the uPA gene and the regions covered by the BAC (RP11-417O11; ∼228 kb, blue) and fosmid (G248P85612C10; ∼44 kb, red) probes used for FISH experiments. Arrows indicate the 5′-3′ transcription direction. (B) The association of BAC and fosmid signals (arrowhead) relative to RNAP-S2p sites (green) was determined simultaneously by immuno-cryoFISH before (unpublished image) and after (B) TPA activation for 3 h, using digoxigenin-labelled BAC (blue) and rhodamine-labelled fosmid (red) probes. High magnification images show examples of the co-association of both BAC- and fosmid-uPA signals with S2p sites (top) or the association of fosmid-uPA signal, but not the BAC signal with S2p sites (bottom). Nucleic acids were counterstained with DAPI (inset). Bar: 2 µm. (C) Frequency of the association of BAC or fosmid signals with S2p sites is similar between probes (χ2 test, p = 0.37 and p = 0.81, n = 46 and 40, for − and +TPA, respectively). Error bars are standard deviations from two replicate experiments. (D) Fosmid signals (red) can loop out of BAC foci (green). Arrowheads indicate the position of BAC and fosmid signals. Insets show higher magnification images. Nucleic acids were counterstained with TOTO-3 (blue). Bar: 2 µm. Frequency of co-localisation of BAC foci with fosmid-uPA signals show that 15%–20% of uPA alleles detected with the fosmid probe loop out from the BAC signals. The difference between the levels of fosmid looping ±TPA was not statistically significant (χ2 test, p = 0.57; n = 47 and 41, for − and +TPA, respectively). (6.78 MB TIF) [file pbio.1000270.s004.tif]

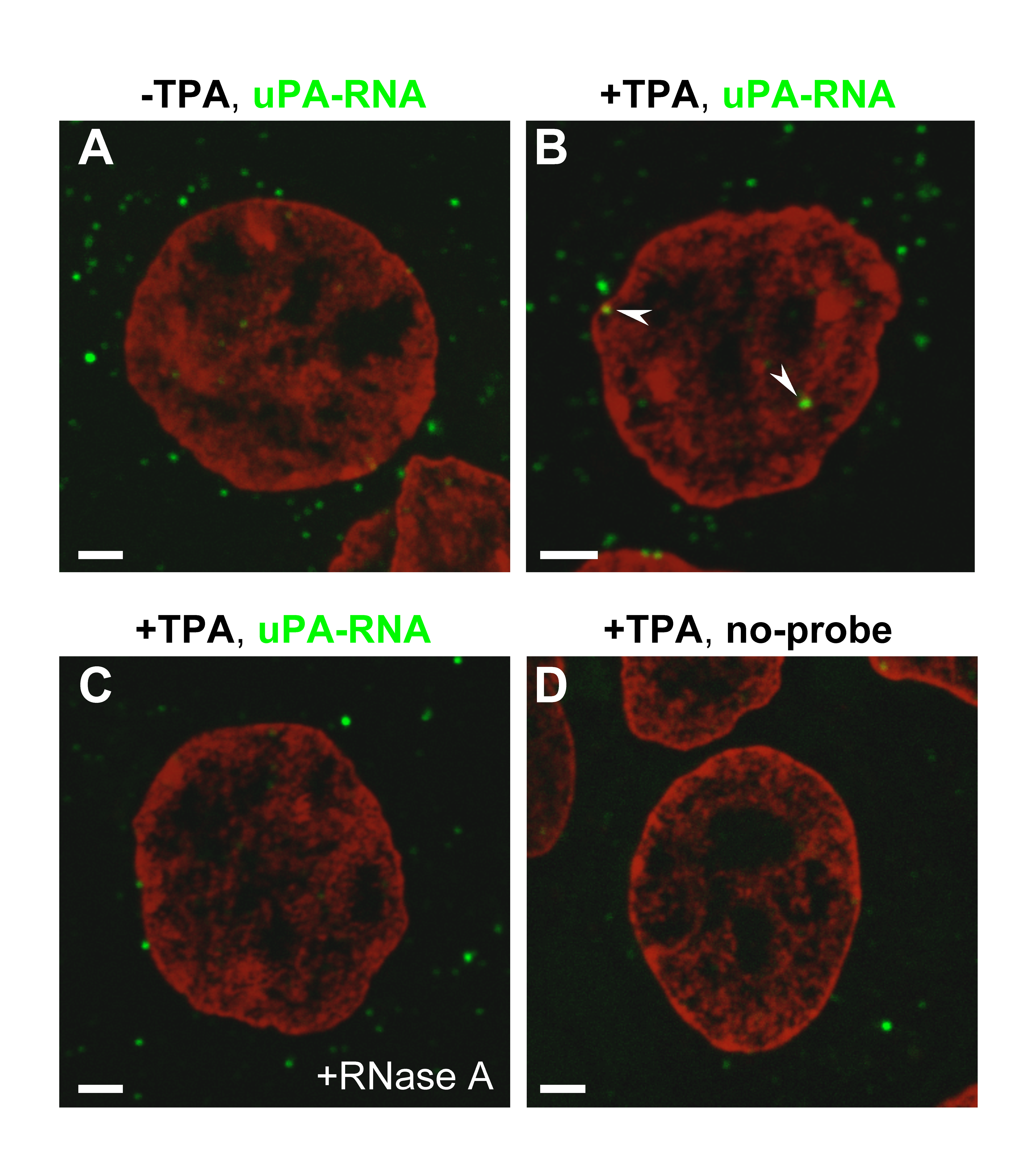

Supplement: Figure S5 — Control experiments for cryo-RNA-FISH. (A–D) Cryosections (∼150 nm thick) of HepG2 cells were hybridised with Cyanine3 labelled uPA oligonucleotide probes before (A) and after (B–D) TPA activation. Inspection of nucleoplasmic regions identifies frequent uPA-RNA signals in TPA-treated cells (B, arrowheads). Pre-incubation of sections with RNase A (C) or omission of oligonucleotide probes (D) abolishes most uPA-RNA signals within the nucleoplasm, demonstrating its specificity. Nucleic acids were counterstained with TOTO-3 (red). Bars: 2 µm. (8.38 MB TIF) [file pbio.1000270.s005.tif]

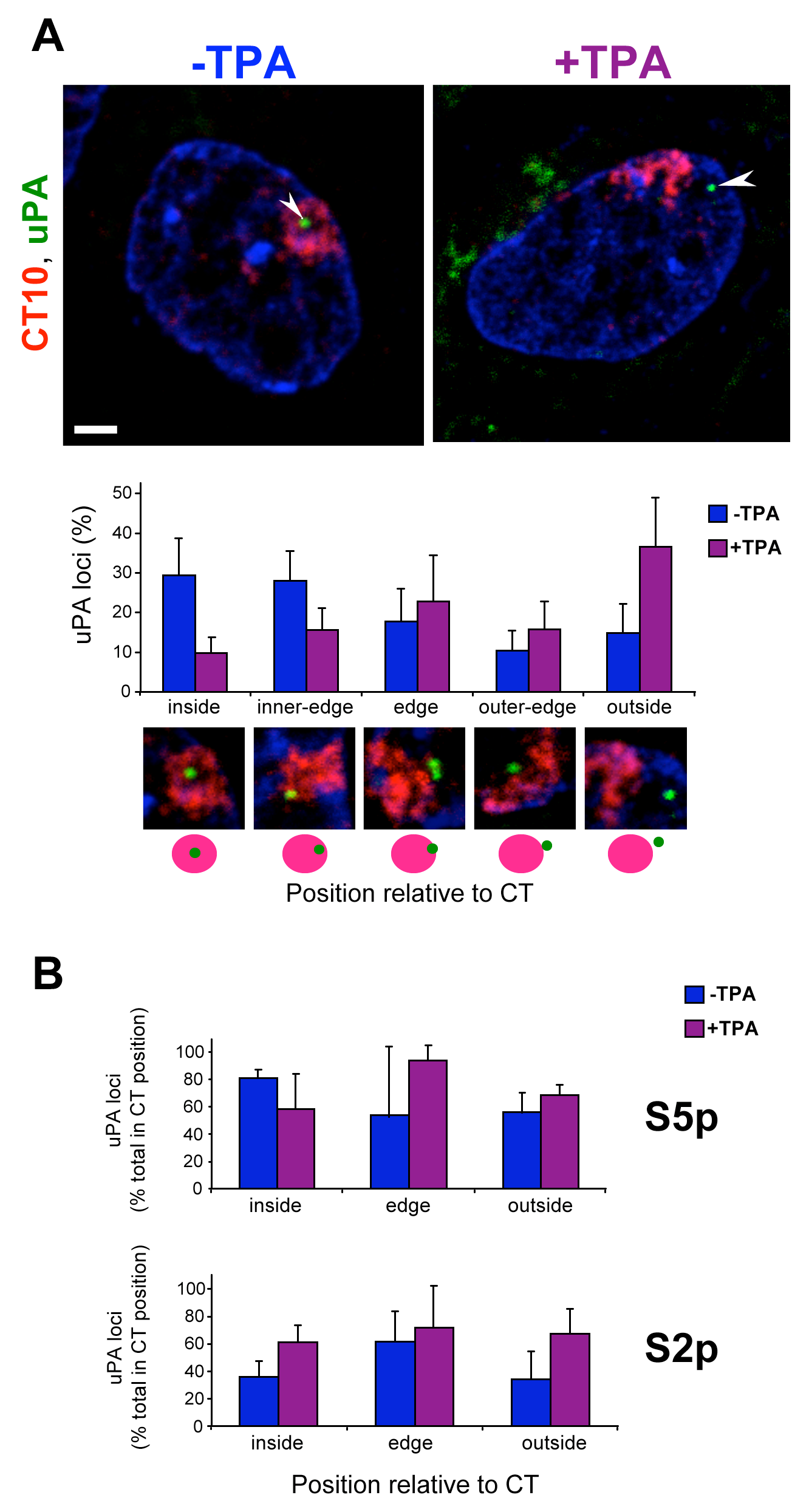

Supplement: Figure S6 — Detection of the uPA gene with a fosmid probe recapitulates the CT looping and position-independent association with S5p and S2p factories observed with the BAC probe. (A) The position of the uPA locus (fosmid-uPA, green) relative to the chromosome 10 territory (CT10, red) was determined in HepG2 cells, before and after TPA activation for 3 h, by cryoFISH using a whole chromosome 10 paint and a digoxigenin-labelled uPA fosmid probe. The positions of uPA loci were scored as “inside,” “inner-edge,” “edge,” “outer-edge,” and “outside” relative to its CT as in Figure 1D. Nucleic acids were counterstained with TOTO-3 (blue). Arrowheads indicate uPA loci. Bar: 2 µm. The histogram shows that the fosmid-uPA probe recapitulates the TPA-induced CT looping observed with the BAC probe (Figure 1D), as expected. In the inactive state, the locus is preferentially localized at the CT interior (61% loci inside or at the inner-edge, n = 234 loci), and relocates to the exterior upon activation (58% loci at outer-edge or outside, n = 230 loci; χ2 test, p<0.0001). (B) The proportion of uPA loci detected using the fosmid probe, which associate with RNAP-S5p before and after TPA activation, was calculated at each CT position (inside, edge, outside) as for the BAC probe (Figure 7C). Association of the locus with S5p sites before and after activation is independent of its position relative to the CT (logistic regression analysis; p = 0.18 and p = 0.26 before and after TPA, n = 68 and 71, respectively). Overall no effect of TPA treatment on the association of the uPA gene with S5p was detected (logistic regression analysis, p = 0.54). Association of uPA loci detected with the fosmid probe with S2p sites before and after activation is also independent of its position relative to the CT (logistic regression analysis, p = 0.27 and p = 0.79 before and after TPA, n = 113 and 140, respectively). This same analysis also detected an increased association of uPA gene with S2p sites upon activation (l [file pbio.1000270.s006.tif]

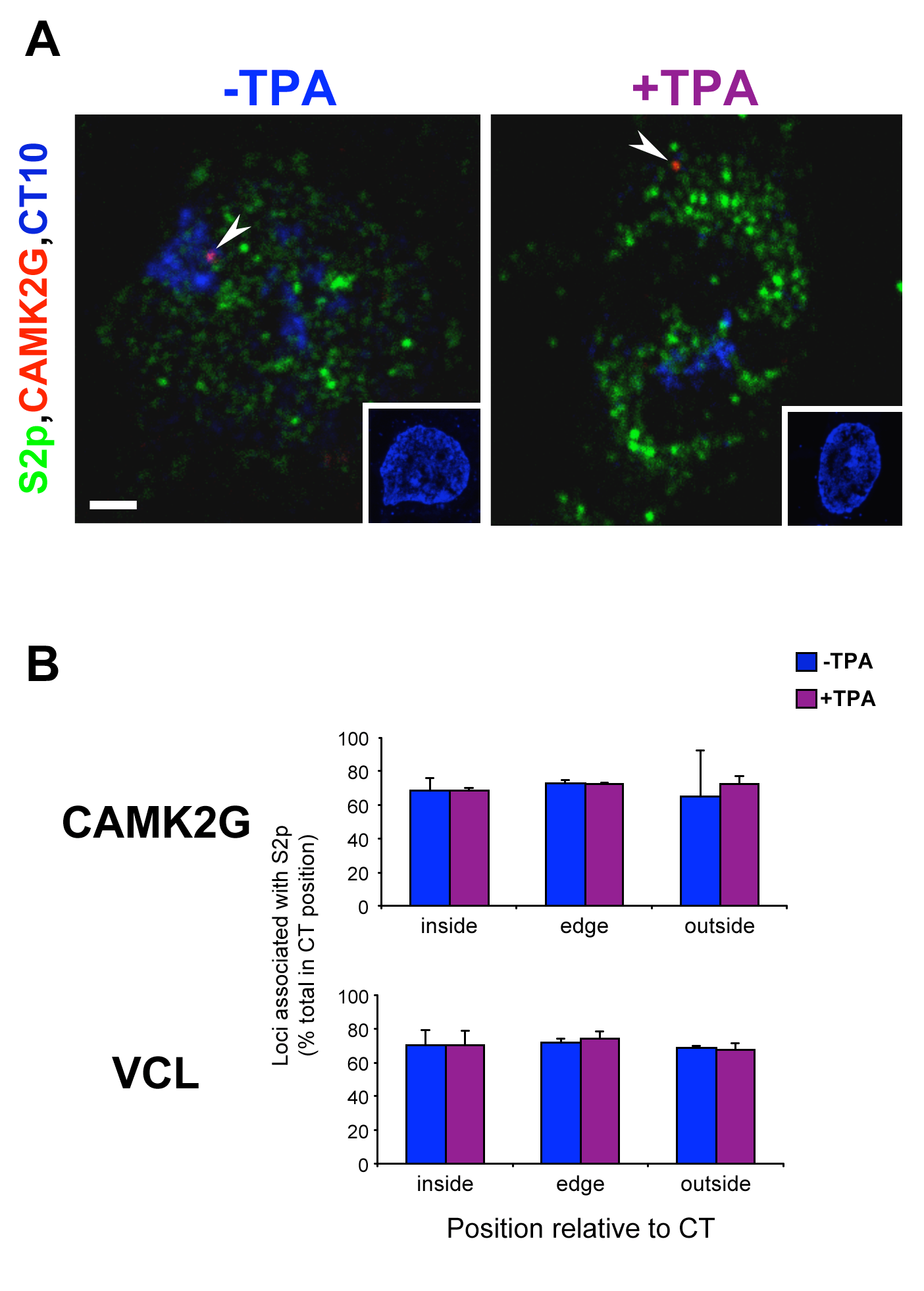

Supplement: Figure S7 — uPA-flanking genes, CAMK2G and VCL, associate with S2p factories independently of CT position or TPA activation. The association of CAMK2G or VCL loci with RNAP-S2p were determined relative to the chromosome 10 territory (CT10) in HepG2 cells, before and after TPA activation (3 h), by cryoFISH using a whole chromosome 10 paint and digoxigenin-labelled CAMK2G and VCL fosmid probes. (A) Images represent examples of CAMK2G loci (red, arrowheads) that co-localise with RNAP-S2p (green) inside (left) or outside (right) of CT10 (blue). Nucleic acids were counterstained with DAPI (insets). Bar: 2 µm. (B) The proportion of CAMK2G or VCL loci, which associate with RNAP-S2p, was determined at each CT position (inside, edge, outside), before and after TPA activation. Association of either gene with S2p is independent of their position relative to the CT and to TPA treatment. (5.98 MB TIF) [file pbio.1000270.s007.tif]

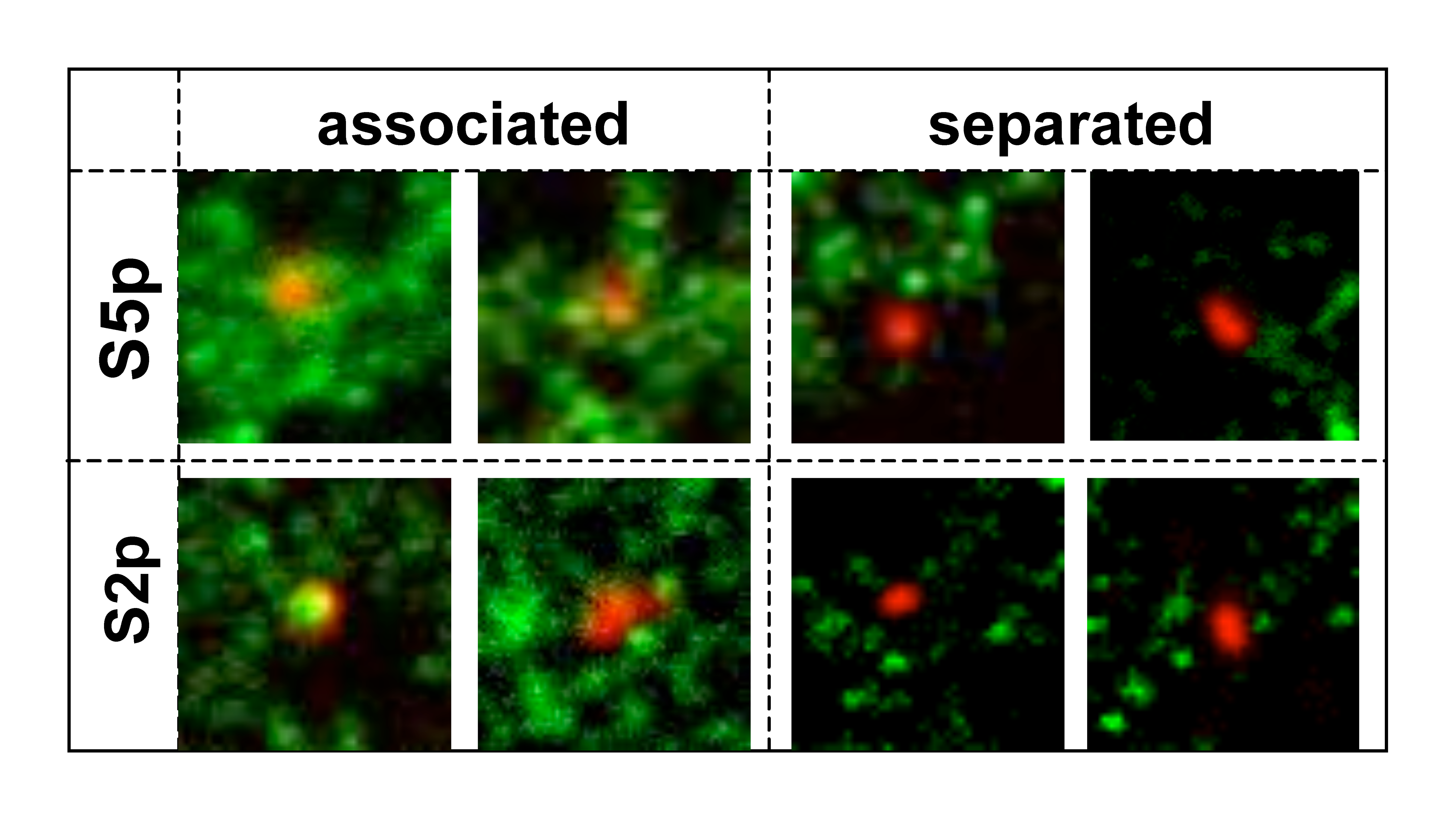

Supplement: Figure S8 — Examples of classification criteria for the association of uPA loci with RNAP-S5p and RNAP-S2p sites. The position of the uPA locus (red) with S5p and S2p sites (green) was determined by immuno-cryoFISH using a rhodamine-labelled BAC probe containing the uPA locus and antibodies specific for RNAP phosphorylated at residues S5 or S2 of the CTD. Associated uPA loci co-localise with S5p or S2p sites if signals overlap by at least a single pixel, whereas separated sites do not show overlap of the two signals and include loci that may touch an RNAP site without signal overlap. (4.11 MB TIF) [file pbio.1000270.s008.tif]
