# Supplementary material for: Poised Transcription Factories Prime Silent uPA Gene Prior to Activation
Source: PLoS Biol. 2010 Jan 5;8(1):e1000270. doi: 10.1371/journal.pbio.1000270 (PMC2797137; doi:10.1371/journal.pbio.1000270)
Supplement: Table S1 — MN-ChIP primers. List of primers used in MN-ChIP analyses in 5′ to 3′ orientation. (0.06 MB DOC) [file pbio.1000270.s009.doc]

Supplementary Table S1. List of primers used in MN-ChIP analyses in 5’ to 3’ orientation.

| Amplification product | Primer sequences (5’-3’) | Primer position in uPA sequence [44] | Annealing temperature  (°C) |
| --- | --- | --- | --- |
| E1 | TGTCCAGGAGGAAATGAAGTCATC  GAAACTCCCAGGTTAGTTATCAGG | -1981/-1958  -1836/-1859 | 57 |
| E2 | TGTCCAGGAGGAAATGAAGTCATC  GACCAGAACATAAACAGAGATGCTG | -1981/-1958  -1792/-1816 | 57 |
| E3 | TGTCCAGGAGGAAATGAAGTCATC  CTCTAGAAGACTGTGGTCAGTTTTG | -1981/-1958  -1731/-1755 | 57 |
| E4 | GGGAGAAAGGGTGTCACGC  GCCGTCATGATTCATGTTGCTCC | -2024/-2006  -1872/-1894 | 57 |
| E5 | GAGGACCCCTTGAACCCAGAAG CCGTGCCACCTCTTCACCTAGC | -2192/-2171  -2043/-2064 | 57 |
| P | GAATTTACAAGCCTCTCGATTCCTC  GGGATCTCAGGACCGCGG | -205/-181  +114/+97 | 60 |
| frag. 1 | GAATTTACAAGCCTCTCGATTCCTC  ACCAGGCTCCCCAGCTGTC | -205/-181  +304/+286 | 60 |
| Px | GAGCTGGGCGAGGTAGAGAGTC  GGGATCTCAGGACCGCGG | -313/-292  +114/+97 | 60 |
| frag. 2 | CAGTAATCTGGCCTTGCCTTTCC  GAGGAATCGAGAGGCTTGTAAATTC | -645/-623  -181/-205 | 60 |
| uP | GAATTTACAAGCCTCTCGATTCCTC  CTCCCTCCCCTGTCTTGCAGC | -205/-181  -65/-85 | 60 |
| dP | GCTGCAAGACAGGGGAGGGAG  GGGATCTCAGGACCGCGG | -85/-65  +114/+97 | 60 |
| H19 Gene | TGATCCCAGGGCCTGGGCAAC  GATGTCACCTTTGCTAACTCTCCT | – | 62 |
