# Supplementary material for: Poised Transcription Factories Prime Silent uPA Gene Prior to Activation
Source: PLoS Biol. 2010 Jan 5;8(1):e1000270. doi: 10.1371/journal.pbio.1000270 (PMC2797137; doi:10.1371/journal.pbio.1000270)
Supplement: Text S1 — Supplementary information. (0.10 MB DOC) [file pbio.1000270.s010.doc]

**Supplementary Experimental Details**

Cell culture and RNA detection

HepG2 cells were cultured in the absence or presence of 100 μM (TPA; Sigma) for 3h, unless otherwise indicated, as previously described [3]. For the inhibition of RNAP Ser2 phosphorylation by CDK9 (Supplementary Figure S2B), HepG2 cells were treated (1h) with 1 μM flavopiridol (from 50 mM stock in DMSO; a kind gift from Sanofi-Aventis, provided by Drug Synthesis and Chemistry Branch, Developmental Therapeutics Program, Division of Cancer Treatment and Diagnosis, National Cancer Institute, Bethesda, MD). HepG2 cells were treated (5h) with 75 μg/ml α-amanitin (Sigma) to inhibit RNAP transcription (Figure 5B).

RNA quantification

For measurements by qRT-PCR of uPA (Figure 1B), CAMK2G and VCL (not shown) expression, total RNA was extracted with RNeasy mini kit (Qiagen) and quantified by spectrophotometry (Nanodrop). Five micrograms of total RNA were reverse-transcribed with random hexamers using a SuperScript^TM^ First-Strand kit (Invitrogen). Five nanograms of reverse-transcribed RNA were amplified and products detected using TaqMan gene expression assays (Applied Biosystems, Foster City, CA) for CAMK2G (Hs00538454_m1), uPA (Hs00170182_m1), VCL (Hs00419715_m1) and for 18S rRNA (4319413E) for normalization, in an ABI PRISM 7900HT Sequence Detection System (Applied Biosystems, Foster City, CA, USA).

For qRT-PCR measurements of transcription levels of uPA, CAMK2G and VCL genes (Figure 5B), total RNA was extracted with Trizol and treated with TURBO DNA-free DNase (Ambion). RNA was reverse transcribed with random hexamers using Superscript II/III kit (Invitrogen), before 20 ng of cDNA product were amplified using SensiMix (Quantace) and products detected using an Opticon 2 machine (BioRad). PCR reactions were performed as follows: first denaturation step: 95°C, 10 min; second denaturation step: 95°C, 30 s; annealing step 60°C, 15 s; extension step: 72°C, 15 s. Primer sequences used (in the 5’-3’ orientation) were:

CAMK2G Fw: AGCCCCGTCTCCTCCTCTTGCTC;

CAMK2G Rev: GCGGGGCGAGAAGCCGCATAGC;

uPA Fw: GCTGGCGCGCCTGCTTCTCTGC;

uPA Rev: TCCCATTTCCTGGGGAGAGTCTGG;

VCL Fw: GGTGATAATGCACGAGGAGGGCG;

VCL Rev: CTCCGGAACCCACGCGGAAAGC.

**MN-ChIP and PCR reactions**

Chromatin cross-linking, MNase digestion and immunoprecipitations were performed as described previously [4]. For the experiment in Figure 2C, genomic DNA was resuspended in distilled water, quantified by spectrophotometry (Nanodrop) and equal amounts (100 ng) were used for each time point as template in PCR reactions. Immunoprecipitation of MNase-digested (50 min) chromatin and subsequent manipulations were carried out as described previously [4]. Resuspended material (4 μl) was used as template in PCR reactions. The antibodies against histone H3 post-translational modifications used for immunoprecipitation were: H3K4me2 (#07-030), H3K9me2 (#07-212), H3K14ac (#07-353) and H3K9ac (#07-352) from Upstate Biotechnology. Monoclonal antibodies against the CTD of RNAP phosphorylated at Ser2 (clone H5; MMS-129-RA) or at Ser5 (H14; MMS-134-RA) residues were from Covance. The polyclonal antibody (M2) against the urokinase-type plasminogen activator receptor (uPAR), used as unrelated antibodies in MN-ChIP experiments, was produced in the Laboratory of Molecular Genetics (Milan) [5].

PCR reactions were performed as follows: first denaturation step: 95°C, 3 min; second denaturation step: 95°C, 1 min; annealing step, 1 min; extension step: 72°C, 1 min; final extension step: 72°C, 3 min. The second denaturation, annealing and extension steps were repeated for 33 cycles. Primer sequences, location with respect to the uPA sequence [2] and annealing temperatures for each primer set are reported in Supplementary Table S1. PCR products were analyzed on 2% (w/v) agarose gels in 1xTAE buffer [4].

**Western blot analysis and antibody validation**

Cells were detached with cold PBS containing 1 mM EDTA and washed with cold PBS. 1x10^6^ cells were resuspended in lysis buffer (50 mM Tris, pH 7.6, 0.8 M NaCl, 1 mM EDTA, 1% Triton X-100, 0.5% NP-40, 1 mM PMSF) containing phosphatase inhibitor cocktail 1 (1:100; Sigma), phosphatase inhibitor cocktail 2 (1:100; Sigma) and protease inhibitors (1:1000; Roche) and lysed by passing them through a 25G needle (Terumo). Lysates were placed on a rocking shaker for 15 min at 4°C, before addition of an equal volume of 2x SDS gel-loading buffer, and heating for 10 min at 95°C. Equal volumes were loaded in a 5-15% gradient SDS-PAGE gel. Western blotting was done as previously described in [1] using the antibodies 4H8 (05-623; Upstate), H14 (MMS-129-RA; Covance) both against RNAP-S5p, H5 (MMS-134-RA; Covance) against RNAP-S2p and N-20 against the N-terminal domain of RNAPII (sc-899; Santa Cruz Biotechnology). Protein loading was normalized using histone H2B antibodies (07-371; Upstate). The specificity of the RNAP antibodies to phospho-epitopes was tested by incubating (1h, 37°C) protein blots with 0.1 U/μl alkaline phosphatase (Roche) in PBS buffer, before decoration with antibodies.

**Ultracryosectioning**

HepG2 cells treated ±TPA were fixed in 4% and 8% paraformaldehyde in 250 mM HEPES (pH 7.6; 10 min and 2h, respectively) as described in [6]. Cell pellets were embedded in 2.1 M sucrose in PBS and frozen in liquid nitrogen. Ultrathin cryosections were cut using an UltraCut UCT 52 ultracryomicrotome (Leica, Milton Keynes, United Kingdom) with ~150 nm in thickness unless otherwise stated, captured in sucrose drops, and transferred to coverslips for cryoimmunolabelling and cryoFISH.

**Immunofluorescence labelling**

For detection of uPA protein expression (Figure 1C), HepG2 cells grown on coverslips, treated ±TPA for 3h and fixed (10 min) with 3% formaldehyde (in 2% sucrose, 1xPBS). After permeabilization **(**5 min**)** with 0.5% Triton X-100 (in 20 mM HEPES pH 7.2, 300 mM sucrose, 50 mM NaCl, 3 mM MgCl_2_), cells were washed (3x) with 0.2% BSA in PBS, incubated (20 min) with 2% BSA in PBS, incubated (30 min) with rabbit antiserum antibodies (1:100; produced in the Laboratory of Molecular Genetics, Milan; in 2% BSA in PBS), washed as above, then incubated (30 min) with the TRITC-labelled secondary antibody against rabbit IgG (Southern Biotech, Birmingham, AL) and washed as above. After counterstaining (5 min) the nuclei with DAPI, cells were washed once and mounted with Immumount (Thermo Scientific) on glass slides. All antibody incubations and washes were carried out at 37^o^C.

For RNAP immunolabelling of cryosections, coverslips were rinsed (3x) in PBS, incubated (30 min) in 20 mM glycine in PBS, rinsed (3x) in PBS, permeabilised (10 min) with 0.1% Triton X-100 in PBS, blocked (30 min) with PBS+ (PBS supplemented with 1% BSA, 0.2% fish skin gelatin, 0.1% casein; pH 7.6), incubated (2h) with primary antibodies against RNAP phosphorylated on S2p (H5; 1:2000; Covance) or S5p (CTD4H8 [1]; 1:3000; Upstate), respectively, in PBS+. After washing (3x, 1h) in PBS+, sections were incubated (1h) with secondary, AlexaFluor488- or AlexaFluor680-conjugated donkey antibodies against mouse IgG (Molecular Probes, Eugene, OR) in PBS+, rinsed (3x, 30 min) in PBS+, and rinsed (3x) in PBS (Supplementary Figure S2C,D). Negative control experiments included omission of primary antibodies (not shown) and treatment (2h; 37^o^C) of cryosections with alkaline-phosphatase (AP) (0.5 U/µl, calf intestinal; New England Biolabs, Hitchin, UK) to test the specificity of antibodies to phosphorylated RNAP by immunolabeling (Supplementary Figure S2E,F). The detection of RNAP sites was optimized by increasing the incubation time and by titrating the antibodies concentration [7]. The highest concentration of the different antibodies yielding only negligible background in sections treated with AP were used for maximal detection of RNAP sites [8]. Coverslips were fixed with 8% PFA in PBS (2h) for cryoFISH.

For antibody blocking experiments (Figure 4D-G), cryosections were first incubated in the presence or absence of the 4H8 antibody (to detect RNAP-S5p) or of an irrelevant blocking antibody (mouse anti-biotin IgG, 1:100; Jackson ImmunoResearch Laboratories) as control. After labelling with a secondary, FITC-conjugated donkey anti-mouse IgG antibody (1:500; Jackson ImmunoResearch Laboratories), sections were fixed (2h) with 8% PFA in PBS to preserve the first immunocomplexes, before incubating with the H5 antibody (to detect RNAP-S2p) and with a secondary, Cyanine3-conjugated donkey anti-mouse IgM antibody (1:2000; µ-chain specific; Jackson ImmunoResearch Laboratories).

**CryoFISH detection of DNA**

CryoFISH was performed directly on fresh cryosections or on cryosections previously immunolabelled with antibodies against RNAP or hybridized with oligonucleotide probes for RNA-FISH and fixed (2h) with 8% PFA in PBS to preserve the immunocomplexes. Sections were first rinsed (3x) in PBS, incubated (15 min) in 20 mM glycine in PBS, rinsed (3x) in PBS, permeabilised (10 min) with 0.2% Triton X-100 and 0.2% saponin in PBS, and then washed (3x) in PBS. Cryosections were incubated (1h, 37^o^C) with 250 μg/ml RNase A (Sigma; in 2xSSC), treated (10 min) with 0.1 M HCl, dehydrated in ethanol (50 to 100% series, 3 min each), denatured (10 min, 80°C) in 70% deionized formamide, 2xSSC, and dehydrated as above. Hybridization was carried out at 37°C in a moist chamber for at least 40h.

The uPA locus was detected using a BAC (RP11-417O1; Figure 1A) or a fosmid probe (G248P85612C10; Figure 5A). The uPA neighbouring genes CAMK2G and VCL were detected with fosmid probes (G248P83711C6 and G248P88122D2, respectively; Figure 5A). Fosmid probes were obtained from BACPAC Resources (California, USA). The specificity of BAC and fosmid probes was confirmed by PCR using specific primers (not shown). Probes were labelled with digoxigenin-11-dUTP, fluorescein-12-dUTP or tetramethyl-rhodamine-5-dUTP by nick translation (Roche), and separated from unincorporated nucleotides using MicroBioSpin P-30 chromatography columns (BioRad, Hertfordshire, UK).

For co-hybridization of uPA and chromosome 10 (Figure 1D, Figure 7 and Supplementary Figures S6 and S7), 5 μg Cot1 DNA (Roche), 10 μg salmon sperm DNA (Sigma) and 5 μl nick-translated uPA BAC or fosmid probes labelled with digoxigenin, respectively, were precipitated and resuspended in 6 μl of chromosome 10 paint (rhodamine-labelled, or biotin-labelled; Applied Spectral Imaging, Israel). For hybridization of the uPA, CAMK2G or VCL loci (Figure 3B,E, Figure 5C,D, Figure 6, Supplementary Figures S3, S4, S8), hybridization mixtures contained 50% deionised formamide (Sigma), 2xSSC, 10% dextran sulphate, 50 mM phosphate buffer (pH 7.0), Cot1 DNA, salmon sperm DNA and the appropriate nick-translated probe. Probes were denatured (10 min) at 80°C and re-annealed (30 min) at 37°C before hybridization.

Posthybridization washes were as follows: 50% formamide in 2xSSC (42°C; 3x over 25 min), 0.1xSSC (60°C, 3x over 30 min), and 0.1% Tween-20 in 4xSSC (42°C, 10 min). Sections were incubated (30 min) with casein-blocking solution (pH 7.8; Vector Laboratories) containing 2.6% NaCl, 0.5% BSA, 0.1% fish skin gelatin.

For double labelling of RNAP and the uPA locus using either a BAC probe (Figures 3B,E), or fosmid probes specific for uPA, CAMK2G or VCL (images not shown; data in Figure 5C,D), the signal of rhodamine-labelled probes was amplified with rabbit anti-rhodamine antibodies (2h; 1:500; Molecular Probes) and Cyanine3-conjugated donkey anti-rabbit IgG (1:1000; Jackson ImmunoResearch Laboratories).

For co-localization of the uPA locus with chromosome territory 10 (Figure 1D and Supplementary Figure S6), uPA probes labelled with digoxigenin were detected (2h) with sheep anti-digoxigenin Fab fragments (1:200; Roche), FITC-conjugated donkey anti-sheep IgG (1:200; Jackson ImmunoResearch Laboratories) and AlexaFluor488 rabbit antibodies anti-FITC (1:500; Molecular Probes, Eugene, OR).

For triple labelling of the uPA locus, RNAP and chromosome 10 (Figure 7A and Supplementary Figure S6B), uPA probes labelled with digoxigenin were detected with sheep anti-digoxigenin Fab fragments (1:100; Roche), biotin-conjugated donkey anti-sheep IgG (1:200; Jackson ImmunoResearch Laboratories) and AlexaFluor647-streptavidin (1:100; Molecular Probes), combined with detection of RNAP-S5p or -S2p with AlexaFluor488 donkey anti-mouse IgG (1:1000; Molecular Probes).

For triple labelling of the CAMK2G and VCL loci, RNAP-S2p and chromosome 10 (Supplementary Figure S7), fosmid probes labelled with Rhodamine or digoxigenin. Rhodamine was amplified as described above for ‘double labelling of RNAP and the uPA locus”. Digoxigenin was detected with sheep anti-digoxigenin Fab fragments (1:100; Roche), FITC-conjugated donkey anti-sheep IgG (1:100; Jackson ImmunoResearch Laboratories). RNAP-S2p was detected with H5 antibody and AlexaFluor488- or AlexaFluor680-conjugated donkey anti-mouse IgG (1:500; Molecular Probes) to combine with either biotin- or rhodamine-labelled chromosome 10 paint accordingly. The biotin-labelled chromosome 10 paint was labelled with streptavidin conjugated with Marina Blue (1:20; Molecular Probes), biotin-conjugated mouse anti-avidin antibodies (1:500; Vector Laboratories) and Marina Blue-conjugated streptavidin (1:40).

For triple labelling of BAC, fosmid uPA loci and RNAP (Supplementary Figure S4B), a digoxigenin-labelled BAC probe and a rhodamine-labelled fosmid probe were detected with AlexaFluor647 and Cyanine3 fluorochomes, respectively, as described above. Nuclei were counterstained (45 min) with 2 μM TOTO-3 (Molecular Probes) or DAPI in PBS with 0.05% Tween-20, rinsed sequentially in 0.05% Tween-20 in PBS and then PBS alone. Coverslips were mounted in VectaShield (Vector Laboratories, Peterborough, UK) immediately before imaging.

**CryoFISH detection of RNA**

The active uPA alleles were detected in cryosections of HepG2 cells using a mixture of five fiftymer oligonucleotide probes that each map at introns 4, 5 and 10, and exons 8 and 9 of the uPA gene, modified with four or five amino-allyl thymidine residues (labelled with an X in the sequences below; Eurogentec S.A. Belgium). Due to the difficulty in the detection of small introns (uPA introns are all <900 bp), which can be promptly removed within seconds of synthesis, we have included two fiftymer probes for exon sequences (see also [9]), to improve the efficiency of detection of transcripts at the site of synthesis. The use of intron probes alone was not sufficient to detect robustly active uPA loci (not shown). Therefore the detection of active uPA genes was performed in all cases by simultaneous detection of the gene and its respective RNA, and not simply by the visualization of RNA-FISH signals alone.

The 50mer oligonucleotide sequences were as follows:

intron 4: 5’-CACTXGTTTCGTGAGCGTXGTGTGTGTGTXTTATGCTTGTXCACATAGGGATXGA-3’;

intron 8: 5’-GTCCCTXCGCCCCACCCCTXGCCCCACCGGGCAGCTXTCAACTCAGCTXCCAGT-3’;

intron 10: 5’-CACCTXGATCATTTATAGTXTCACAACACACCCTXGGGCACTXGAGTTACATXTG-3’;

exon 8: 5’-CAAACTXTCATCTCCCCTXTGCGTGTXTGGAGTTXAAGCCTTXGAGCGACCCAGG-3’;

exon 9: 5’-CAGATXGGTCTGTATAGTXCCGGGATGGCTXGCGCACACCTXGCCCTCCTTXGGA-3’;

Each oligonucleotide was labeled with Cyanine3 mono-reactive dye, essentially as described previously (see: http://www.singerlab.org/protocols). Briefly, 10 μg modified oligonucleotide (100 μM in H_2_O stock solution) were diluted in 70 μl carbonate buffer (0.1M, pH 8.8), and mixed with 1 vial of monofunctional Cyanine3 labelling kit (GE Healthcare Life Sciences) previously resuspended into 30 μl DMSO. After incubation (overnight, 20^o^C, in the dark with shaking), the conjugated oligonucleotides were separated from unconjugated dye by two consecutive ethanol precipitations.

RNA-FISH was performed essentially as previously described (see: http://www.singerlab.org/protocols) and adapted for cryosections as follows. Freshly cut cryosections (Figure 6A, Figure 7D and Supplementary Figure S5) or cryosections previously indirectly immunolabelled with antibodies against S2p and AlexaFluor488 (Figure 6B), were first rinsed (3x) in diethyl pyrocarbonate-treated PBS (DEPC-PBS), incubated (15 min) in 20 mM glycine in DEPC-PBS, rinsed (3x) in DEPC-PBS, permeabilised (10 min) with 0.2% Triton X-100 in DEPC-PBS containing 2 mM Vanadyl Ribonucleoside Complex (VRC, Sigma), and then washed (3x) in DEPC-PBS. Cryosections were then rinsed with DEPC-PBS containing 5mM MgCl_2_  (DEPC-PBSM), dehydrated in 30, 50 and 70% ethanol series (3 min each; 20^o^C) and stored in 70% ethanol for <16h. After rinsing in DEPC-PBSM and incubation (15 min) with 20% formamide in 1xSSC, hybridizations were carried out at 37°C in a moist chamber for at least 40h. Hybridization mixtures consisted of 20% deionised formamide, 1xSSC, 170 ng/μl tRNA (Roche), 10% dextran sulphate, 1% BSA, 2 mM VRC and 2 ng of each Cyanine3-labelled oligonucleotide. Hybridization mixtures were denatured at 95°C for 1 min before hybridization. After hybridisation, sections were post-washed (30 min, 37°C) with 20% formamide in 1xSSC, rinsed in DEPC-PBSM and then incubated (30 min) with PBS+, as previously indicated for immunodetection. The Cy3-uPA RNA signal was amplified by incubating with sheep anti-cyanine antibodies (1:100; Abcam, Cambridge, UK) and Alexafluor555-conjugated donkey anti-sheep IgG (1:1000; Molecular Probes). Control experiments included omission of probe (Supplementary Figure S5D) or treatment (2h; 37^o^C) of cryosections with RNaseA (250 μg/ml) before hybridization (Supplementary Figure S5C) to show the specificity of uPA RNA detection.

For co-localization of the uPA locus with uPA RNA (Figure 6A), the uPA fosmid probe labelled with digoxigenin was detected with AlexaFluor488-conjugated antibody as before. For triple labelling of the uPA locus, uPA RNA and RNAP (Figure 6B), the uPA fosmid probe labelled with digoxigenin was detected with an AlexaFluor647 conjugated antibody, as described above in the “cryoFISH detection of DNA” section.

For triple labelling of the uPA locus, active uPA RNA gene and chromosome 10 (Figure 7D), the uPA fosmid probe was labelled with fluorescein, the CT probe was labelled with biotin, and the uPA RNA with Cyanine3 oligonucleotides. The signal of the uPA fosmid probe labelled with fluorescein was amplified with AlexaFluor488-conjugated rabbit anti-FITC IgG (1:500), rabbit anti-AlexaFluor488 (1:1000) and AlexaFluor488 conjugated donkey anti-rabbit IgG (1:1000; all Molecular Probes). The biotin-labelled CT probe was amplified with streptavidin conjugated with Marina Blue as described above in the “detection of DNA cryoFISH” section . Nuclei were counterstained with TOTO-3 or DAPI and coverslips mounted in VectaShield.

**Microscopy and Image Analysis**

Images in Figure 1B were acquired with a confocal laser-scanning microscope (Leica) TCS SP2 (63x Plan-Apochromat oil immersion objective, NA 1.4), equipped with HeNe (405/543 nm) lasers, using a pinhole equivalent to 1 Airy disk at the Advanced Light and Electron Microscopy BioImaging Centre (A.L.E.M.B.I.C.), S. Raffaele Scientific Institute, Milan, Italy.

Images from cryosections were acquired on a confocal laser-scanning microscope (Leica TCS SP5; 60x objective, NA 1.4) equipped with a 405 nm diode, and Argon (488 nm), HeNe (543 nm) and HeNe (633 nm) lasers, using pinhole equivalent to 1 Airy disk. Images from the different channels were collected sequentially to prevent fluorescence bleedthrough.

Images (TIFF files) were merged in Adobe Photoshop, and contrast stretched without thresholding.

For antibody blocking experiments, imaging conditions were calibrated on the positive control samples without saturation of the intensity signal, and collected using the same settings on the same day. Raw TIFF images were merged in Adobe Photoshop, without any further thresholding, contrast stretching or filtering. The intensity of fluorescence in the nucleoplasm was determined in Photoshop (average intensity across the nucleoplasmic area) and subtracted from the background signal in the same image (from an area in the same image not containing section profiles). The association of uPA genes with RNAP (S2p or S5p) was scored as ‘associated’ (signals overlap at least 1 pixel) and ‘separated’ (signals do no overlap; including adjacent signals; see Supplementary Figure S8). The spatial relationship between uPA loci and chromosome 10 was scored as ‘inside’, ‘inner edge’, ‘edge’, ‘outer edge’ and ‘outside’ of the chromosome territory. uPA loci at the ‘inner edge’ touched the surface of the CT with most pixels contained inside the territory. For loci at the ‘outer edge’, most pixels were found outside the territory. Loci at the CT ‘edge’ had approximately half the signal inside and the other half outside.

**Generation of randomly positioned uPA loci**

Experimental images collected for the analysis of uPA locus position relative to transcription factories were used for the generation of randomly positioned (simulated) loci (Supplementary Figure S3B). We developed a macro on ImageJ (Wayne Rasband, NIH, USA) that first thresholds the nuclear counterstaining signal to generate a mask for each nuclear section. Each signal from the BAC and fosmid probes labelling of the uPA gene was also thresholded to estimate their sizes (average 586 and 353 nm diameter for fosmid and BAC probes, respectively). Random *x* and *y* coordinates were then generated, until a circle with the same area as each BAC or fosmid probe signal fell fully within the mask of the corresponding nuclear section, after which the ‘virtual’ signal area was filled to give a randomly positioned locus. Loci that fell within nucleoli (defined by a relatively large nucleoplasmic area containing weak nuclear counterstaining but no RNAP factories) were discarded and generated again. The images containing computer-generated loci were scored for association with transcription factories as before.

**Statistical analyses**

Weighted averages and standard deviations were calculated from 2-4 independent experiments, taking into account sample size in each experiment. Data from replicate coverslips within the same experiment (at least two in most experiments) were collated. Statistical analyses were performed using χ^2^ test and logistic regression analysis in SAS software (v9.1). For Figures 4C the four datasets were analysed by factorial analyses of variance (ANOVA) in SAS and the estimates of the effects were used in pair-wise Student t test. Assumptions of the ANOVA model were confirmed by graphical diagnostic tests. The Mann-Whitney U test was used for the analyses of qRT-PCR represented in Figure 5B.

**Supplementary References**

1. Stock JK, Giadrossi S, Casanova M, Brookes E, Vidal M, et al. (2007) Ring1-mediated ubiquitination of H2A restrains poised RNA polymerase II at bivalent genes in mouse ES cells. Nat Cell Biol 9: 1428-1435.

2. Verde P, Boast S, Franze A, Robbiati F, Blasi F (1988) An upstream enhancer and a negative element in the 5' flanking region of the human urokinase plasminogen activator gene. Nucleic Acids Res 16: 10699-10716.

3. Ibañez-Tallon I, Caretti G, Blasi F, Crippa MP (1999) In vivo analysis of the state of the human uPA enhancer following stimulation by TPA. Oncogene 18: 2836-2845.

4. Ferrai C, Munari D, Luraghi P, Pecciarini L, Cangi M, et al. (2007) A transcription-dependent MNase-resistant fragment of the uPA promoter interacts with the enhancer. J Biol Chem 282: 12537-12546.

5. Resnati M, Pallavicini I, Daverio R, Sidenius N, Bonini P, et al. (2006) Specific immunofluorimetric assay detecting the chemotactic epitope of the urokinase receptor (uPAR). J Imm Met 308: 192-202.

6. Guillot PV, Xie SQ, Hollinshead M, Pombo A (2004) Fixation-induced redistribution of hyperphosphorylated RNA polymerase II in the nucleus of human cells. Exp Cell Res 295: 460-468.

7. Branco MR, Xie SQ, Martin S, Pombo A (2006) Correlative microscopy using Tokuyasu cryosections: applications for immunolabeling and in situ hybridization. In: Stephens D, editor. Cell Imaging. Bloxham, UK: Scion Publishing. pp. 201-217.

8. Xie SQ, Martin S, Guillot PV, Bentley DL, Pombo A (2006) Splicing speckles are not reservoirs of RNA polymerase II, but contain an inactive form, phosphorylated on serine2 residues of the C-terminal domain. Mol Biol Cell 17: 1723-1733.

9. Levsky JM, Shenoy SM, Pezo RC, Singer RH (2002) Single-cell gene expression profiling. Science 297: 836-840.
